# Bridging the Artificial Intelligence Translation Gap in Perioperative Pulmonary and Respiratory Complications: A Practical Framework for Institution-Level Implementation

**DOI:** 10.3390/diagnostics16193082

**Published:** 2026-09-23

**Authors:** Ji-Yeon Lee, Yong-Ho In, Jong-Ho Kim, Sung-Mi Hwang, Jae-Jun Lee, Young-Suk Kwon

**Affiliations:** 1 Big Data Center, Chuncheon Sacred Heart Hospital, College of Medicine, Hallym University, Chuncheon 24253, Republic of Korea; jylee@hallym.or.kr (J.-Y.L.); poik99@hallym.or.kr (J.-H.K.); 2 Institute of New Frontier Research, College of Medicine, Hallym University, Chuncheon 24253, Republic of Korea; inyh66@hallym.ac.kr; 3 Department of Anesthesiology and Pain Medicine, Chuncheon Sacred Heart Hospital, College of Medicine, Hallym University, Chuncheon 24253, Republic of Korea; h70sm@hallym.or.kr

**Keywords:** artificial intelligence, machine learning, postoperative complications, perioperative period, respiratory insufficiency, risk assessment, translational research

## Abstract

Perioperative pulmonary and respiratory complications (PPRCs) result in increased morbidity, mortality, and healthcare costs. Recent research has focused on employing artificial intelligence (AI) and machine learning to predict and analyze PPRCs using expansive perioperative datasets. Despite advances in feature engineering, deep learning architectures, and explainable AI, models are rarely implemented in routine clinical practice. In this narrative review, we synthesize evidence from 59 unique publications, corresponding to 63 phenotype-specific study-listings across six PPRC phenotypes (combined postoperative pulmonary complications, pneumonia/pulmonary infection, hypoxemia, respiratory failure, pulmonary embolism/venous thromboembolism, and pulmonary edema) and introduce an integrated analytical and translational framework to bridge translation gap. The proposed paradigm comprises a tiered framework for evaluating outcome ascertainment quality (TIER) (39 of 63 study-listings [61.9%] were classified as TIER A according to the proposed framework), an algorithm–cohort–phenotype fit model, a discrimination–calibration–utility triad for performance assessment, a translation gap framework highlighting interacting challenges across validation, deployment, and evidence generation, and a six-step framework for clinical translation. Most models lacked robust validation, with only 9 of the 59 unique publications (15.3%) reporting true cross-institutional external validation, thereby limiting their generalizability. Near-term clinical translation is more likely through institution-specific development and local adaptation than direct widespread deployment, supported by concrete recommendations for clinicians.

## 1. Introduction

Postoperative complications increase morbidity, mortality, and healthcare costs by prolonging hospital stays and necessitating additional treatment [1,2]. Postoperative pulmonary complications (PPCs) affect 5–10% of patients undergoing non-thoracic surgery and up to 22% of high-risk individuals [3], with higher 30-day mortality after major surgery [4]. Clinically significant respiratory events extend beyond the postoperative period: intraoperative hypoxemia occurs in 6–8% of surgical patients, and acute pulmonary embolism (PE) may arise during or shortly after surgery [5,6,7], potentially leading to cardiac arrest and perioperative mortality.

Preventing perioperative pulmonary and respiratory complications (PPRCs) requires preoperative risk–based optimization, intraoperative protective strategies, and proactive postoperative management [8]. Validated risk scores such as Assess Respiratory Risk in Surgical Patients in Catalonia (ARISCAT) and Local Assessment of Ventilatory Management during General Anesthesia for Surgery (LAS VEGAS) [9] fail to provide personalized estimates and capture real-time intraoperative changes. As events like hypoxemia and PE evolve dynamically during surgery [10,11,12], static preoperative tools are often insufficient, underscoring the need for data-driven, time-sensitive predictive models.

Predictive models based on artificial intelligence/machine learning (AI/ML) and built using large-scale clinical data are increasingly being applied to PPRC risk assessment [13]. However, clinical adoption is limited by heterogeneity in outcome definitions and observation periods, lack of external validation, integration challenges, and insufficient explainability. In this review, we define “AI/ML-based predictive modeling” as supervised or unsupervised learning applied to perioperative outcome prediction, classification, and feature selection [14].

Reviews have broadly examined perioperative AI or pulmonary and respiratory complications from a conventional clinical perspective [15,16,17,18,19]; however, to our knowledge, no previous review has specifically integrated AI research related to PPRCs (including intraoperative and postoperative outcomes) with practical strategies for local adaptation and institution-level implementation.

In this narrative review, we address this gap by synthesizing AI research across distinct PPRC phenotypes, identifying recurring methodological and translational limitations, and proposing a practical framework for local adaptation and institution-level implementation of AI in perioperative clinical practice.

## 2. Materials and Methods

### 2.1. Search Strategy

To ensure comprehensive coverage across distinct clinical entities, we developed phenotype-specific search strategies combining medical subject headings (MeSH) and free-text terms for six PPRC categories: (1) combined PPCs, (2) postoperative pneumonia and pulmonary infection, (3) perioperative hypoxemia, (4) postoperative respiratory failure, (5) PE, and (6) postoperative pulmonary edema. The eligibility criteria and detailed search strategies for each category are provided in Tables S1 and S2.

The reference lists of included articles and relevant review papers were manually screened to identify additional eligible studies. The overall study-selection process, from phenotype-specific literature searches and screening to the final inclusion of 59 unique publications contributing 63 phenotype-specific study-listings, is summarized in Figure 1.

#### 2.1.1. Search Dates

We conducted a structured literature search to identify studies involving AI- and ML-based approaches to PPRCs. PubMed and Google Scholar were searched in December 2025 for English-language articles. The search covered literature published from January 2018 onward, and studies identified during the search were screened for eligibility. Articles identified during the December 2025 search were retained if they met the eligibility criteria, including those available online at the time of the search but assigned a final bibliographic publication year of 2026. The exact day of search execution was not prospectively recorded.

#### 2.1.2. PubMed Search Methodology

For PubMed searches, we applied Boolean combinations of MeSH terms and free-text keywords using field tags [tiab] and [Title/Abstract] across phenotype-specific queries. Full search strings for both sources are documented in Table S2 for transparency and reproducibility of the search approach.

#### 2.1.3. Google Scholar Search Methodology and Screening

Given the limitations of Google Scholar for systematic retrieval, such as the absence of controlled vocabulary such as MeSH, reduced Boolean functionality, and a ranking algorithm that prioritizes citation impact, we used Google Scholar as a complementary source rather than a primary retrieval tool. For each phenotype-specific search, the first 200 results ranked by Google Scholar relevance were screened. This pragmatic cutoff was used because Google Scholar returns large numbers of increasingly less relevant results and does not provide the reproducible structured retrieval functions available in bibliographic databases. When available, bibliographic information for records identified through Google Scholar was cross-checked against PubMed.

### 2.2. AI/ML Definition and Eligibility Criteria

Our definition of AI/ML encompassed classical supervised learning and modern ML/deep learning, consistent with clinical prediction modeling literature. Studies were included if they explicitly framed their methodology as ML- or AI-based, regardless of the specific algorithm used. Perioperative care encompassed surgical procedures under general, regional, or monitored anesthesia and non-surgical procedures requiring procedural sedation, reflecting the breadth of clinical contexts in which PPRCs may occur (Table S1). Eligibility was independently assessed by two reviewers with experience in clinical data research. Discrepancies were discussed and resolved by consensus. Given the narrative nature of this review, no formal risk-of-bias assessment was applied; instead, methodological characteristics were qualitatively appraised within each phenotype, focusing on validation strategy, outcome definitions, feature sets, and performance metrics (see Figure 2).

### 2.3. Development and Scope of the TIER Framework

TIER is a pragmatic, review-specific descriptive framework focused exclusively on outcome ascertainment. TIER was developed for grading the rigor of outcome ascertainment processes in the PPRC literature. Although the tiered classification scheme was developed for this review, its underlying methodological principles are informed by established outcome-related concepts in Transparent Reporting of a multivariable prediction model for Individual Prognosis or Diagnosis (TRIPOD) + AI and Prediction model Risk-Of-Bias Assessment Tool (PROBAST)/PROBAST + AI [20,21,22]. TIER does not assess over-all risk of bias, applicability, or reporting quality, and calibration assessment and decision-curve analysis are not components of the TIER classification. Regarding local implementation, TIER may serve as a reference for institutions assessing the reproducibility of outcome definition and ascertainment process of an existing model in their local setting. However, TIER should not be interpreted as an indicator of overall model quality, transportability, or clinical utility, which require separate evaluation.

- TIER A (higher-rigor ascertainment): adjudicated clinical review, imaging-confirmed diagnosis where appropriate, or validated composite definitions incorporating explicit clinical criteria;
- TIER B (intermediate-rigor ascertainment): validated administrative algorithms, natural language processing (NLP)-derived outcomes with reported performance, or explicitly defined clinical criteria without higher-level confirmation where such confirmation would otherwise be applicable; and
- TIER C (basic ascertainment): unvalidated administrative coding alone, self-report, or insufficiently specified/ambiguous outcome definitions.

Throughout this review, we distinguish between “unique publications” (*n* = 59), referring to distinct published articles, and “study-listings” (*n* = 63), referring to phenotype-specific entries derived from these publications for the evidence synthesis. A publication evaluating more than one PPRC phenotype contributed a separate study-listing for each applicable phenotype. TIER classification was assigned at the study-listing level because outcome definition and ascertainment could differ across PPRC phenotypes within the same publication.

Study-level classifications, including TIER category, validation type, and algorithm category, were initially assigned by one investigator and subsequently reviewed by a second. Discrepancies were discussed and resolved by consensus.

### 2.4. Use of AI-Assisted Tools

Large language models (LLMs) were used as supportive tools during manuscript preparation and revision. During the initial drafting phase, GPT-4o was used to assist with English-language formulation, visualization of planned figures, and summarization of Supplementary Tables. During the revision phase, Claude Opus was used to assist with restructuring the manuscript outline, methodological framing, and iterative review of section coherence and clarity. These tools were used solely for drafting, language, organizational, and editorial support. All literature screening, data extraction and classification, methodological decisions, interpretation of the evidence, and final revisions were performed or verified by the human authors. All AI-assisted outputs incorporated into the manuscript were critically reviewed, edited, and verified by the authors.

## 3. Scope of PPRCs

The American College of Surgeons National Surgical Quality Improvement Program (ACS-NSQIP) routinely monitors 16 major postoperative complications [23,24], including pulmonary- and respiratory-specific events such as unplanned intubation, ventilator dependence > 48 h, PE, and pneumonia. However, few studies have deployed AI to predict unplanned intubation or prolonged ventilation as defined by ACS-NSQIP. To provide a comprehensive overview, we included studies predicting postoperative respiratory failure (PRF) and acute respiratory distress syndrome (ARDS), which share similar pathophysiology and respiratory deterioration, and are frequently used as surrogate endpoints in perioperative research [25]. PE is among the most clinically consequential PPRCs, yet postoperative AI/ML studies modeling PE as a standalone outcome are scarce. Accordingly, this review includes studies addressing PE within broader venous thromboembolism (VTE) outcomes, such as deep vein thrombosis (DVT) and PE reported separately within multi-endpoint frameworks or VTE composite (DVT + PE merged).

Beyond outcomes defined by ACS-NSQIP, other clinically important PPRCs include pulmonary infection, pulmonary edema, and POH. Hypoxemia does not only occur during surgery under general anesthesia but also during procedural sedation, where preserved spontaneous ventilation introduces distinct pathophysiological considerations. Although these events are not consistently captured in ACS-NSQIP data, they represent meaningful real-world complications affecting patient prognosis and perioperative management [26,27,28]. This review includes studies addressing combined PPCs, ensuring coverage of the full spectrum of PPRCs explored in AI-based modeling research.

The broad clinical spectrum of PPRCs can be approached either as a composite target, as in studies of combined PPCs, or as a collection of distinct perioperative phenotypes with different temporal characteristics, clinical consequences, and opportunities for intervention. Accordingly, AI-related evidence should be interpreted within the clinical context and the specific composite outcome or individual phenotype being addressed. This perspective provides the clinical basis for examining AI research across combined PPCs and individual PPRC phenotypes in the sections below.

## 4. AI Models by PPRC Phenotype

### 4.1. Combined PPCs

Of the 63 phenotype-specific study-listings, 10 addressed combined PPCs (Table 1 and Table 2 and Table S3) [29,30,31,32,33,34,35,36,37,38]; six (60%) of the highest consensus framework adoption rates across the six phenotypes have been discussed in this review.

Seven studies reported values for area under the receiver operating characteristic curve (AUROC) > 0.8 [29,32,33,35,36,37,38]; however, only one study conducted external validation using independent institutional data [35]. Two studies [29,34] employing prospective internal temporal validation showed substantial performance degradation, suggesting that internal validation alone systematically overstates real-world generalizability.

Two mechanistic limitations warrant explicit attention. First, individual complication incidence varies widely within composite outcomes. Common events such as radiologically detected atelectasis dominate composite endpoints, whereas clinically severe but infrequent complications (PE, ARDS) contribute minimally to model training. Algorithms may therefore be biased toward high-incidence and low-severity events, and combining heterogeneous complications with differing pathophysiology and prognostic impacts into a single category limits discriminative power and external validity. Second, key predictor variables such as American Society of Anesthesiologists physical status, estimated blood loss, and pulmonary function tests are often inconsistently recorded across institutions, further limiting cross-institutional implementation.

### 4.2. Postoperative Pneumonia and Pulmonary Infection

Overall, 17 studies (17/63, 27%) developed models for pneumonia [24,39,40,41,42,43,44,45,46,47,48,49,50,51,52,53,54] while 4 (4/63, 6.3%) addressed pulmonary infections [55,56,57,58], a broader endpoint that may include pneumonia and other lower respiratory tract infections (Table 1 and Table 2 and Table S4). Definitions of postoperative pneumonia varied across studies, but most incorporated clinical symptoms, laboratory evidence of infection, and radiological findings. Three of the four studies addressing pulmonary infections reported explicit criteria [56,57,58]. Observation windows ranged from 48 h postoperatively to the entire hospital stay, with the 48 h restriction more conservative than standard clinical practice [42], potentially increasing diagnostic specificity by capturing complications closely related to the surgical episode and identifying cases with greater early-prevention potential.

Heterogeneity in outcome definitions and cohort characteristics limits direct comparison across studies. However, unlike studies on combined PPC models, most studies on pneumonia and pulmonary infections use similar core diagnostic components.

**Table 1:** Overview of AI/ML models for perioperative pulmonary and respiratory complications across six phenotypes (63 study-listings): validation strategy classification.

Phenotype	*n*	Geographic Distribution	Institution-Led Multicenter	Registry-Based	Single-Center	External Validation	TIER A	Notable Features (AUROC Range)
Combined PPCs	10	China 7, South Korea 1, BioStudies 2	3 (30.0%)	2 (20.0%)	5 (50.0%)	1 (10.0%)	60%	AUROC 0.67–0.89; 60% consensus framework (highest); 2 prospective ITV
Pneumonia/pulmonary infection	21	China 15, South Korea 2, US 2, China + MIMIC IV 1, ACS-NSQIP 1	4 (19.0%)	1 (4.8%)	16 (76.2%)	3 (14.3%)	8/21 (38.1%) consensus or comparable	AUROC 0.72–0.94 (excluding overfitting signature 0.998); 5/21 LR-based nomograms (23.8%)
Hypoxemia	8	South Korea 4, China 3, US 1	3 (37.5%)	0 (0%)	5 (62.5%)	2 (25.0%)	100% (objective SpO_2_)	AUROC 0.73–0.94; 75% tree-based, 25% hybrid/mixed (4-model with LR [59]); 2 ITV
Respiratory failure	12	US 3, US registries 4, China 4, South Korea 1	3 (25.0%)	4 (33.3%)	5 (41.7%)	3 (25.0%)	TIER A 58.3% (Berlin ARDS, STEP-COMPAC, and other consensus frameworks)	AUROC 0.61–0.93; 0% LR/linear (physician-dependent outcomes)
Pulmonary embolism/venous thromboembolism	11	US 4, China 3, ACS-NSQIP 2, MBSAQIP 2	1 (9.1%)	4 (36.4%)	6 (54.5%)	2 (18.2%)	81.8% (Clavien-Dindo 9%)	AUROC 0.60–0.91 (excluding overfitting signature 0.982); most diverse algorithms; 3 ITV
Less-studied (pulmonary edema)	1	South Korea 1	1 (100%)	0 (0%)	0 (0%)	0 (0%)	TIER A (radiologist CXR)	Balanced RF (AUROC 0.91, PR-AUC 0.44); multicenter IV
Total	63	China 33 (incl. MIMIC IV 1), US 10, South Korea 9, US Registries 9, BioStudies 2	15 (23.8%)	11 (17.5%)	37 (58.7%)	11 (17.5%)	61.9% (39/63)	AUROC 0.60–0.94 (excluding overfitting signatures); 7 nomograms (6/7 LR-based)

Multicenter classification: Institution-led multicenter = single institution or partnership leading data collection across multiple hospital sites within a defined consortium; registry-based = studies using national or international registries inherently multi-institutional by design (ACS-NSQIP, MBSAQIP, BioStudies, National Inpatient Sample, Nationwide Readmissions Database, NEPHRON pediatric registry); single-center = single institution data collection. External Validation = validation using an independent institutional dataset distinct from the training data source. Critically, registry-based design does not equate to external validation; cross-institutional EV using independent institutional datasets remains the gold standard for assessing model generalizability. TIER A = outcome ascertainment based on international consensus criteria or objective measurement; TIER B = national/society guidelines or study-specific definitions with explicit criteria; TIER C = ICD codes or implicit definitions (framework grounded in TRIPOD + AI reporting guideline and PROBAST risk-of-bias assessment tool). ITV = internal temporal validation. Prospective ITV represents a robust internal validation strategy using temporally distinct cohorts (development cohort followed by chronologically later validation cohort within the same institution(s)). Abbreviations: ACS-NSQIP = American College of Surgeons National Surgical Quality Improvement Program; AI/ML = artificial intelligence/machine learning; ARDS = acute respiratory distress syndrome; AUROC = area under the receiver operating characteristic curve; CXR = chest X-ray; EV = external validation; ICD = International Classification of Diseases; ITV = internal temporal validation; IV = internal validation; LR = logistic regression; MBSAQIP = Metabolic and Bariatric Surgery Accreditation and Quality Improvement Program; MIMIC IV = Medical Information Mart for Intensive Care IV; PE = pulmonary embolism; PR-AUC = precision–recall area under the curve; RF = random forest; SpO_2_ = peripheral oxygen saturation; TIER = tiered framework for outcome ascertainment quality; VTE = venous thromboembolism.

### 4.3. Perioperative Hypoxemia

Eight studies (8/63, 12.7%) addressed POH (Table 1 and Table 2 and Table S5) [59,60,61,62,63,64,65,66]. All POH definitions were based on objective peripheral oxygen saturation criteria, with thresholds ranging from <90% to <95%. The threshold choice has direct clinical implications; values < 90% correspond to clinically significant hypoxemia requiring intervention, whereas values > 90% may serve as early warning indicators. Desaturation duration is also important, as models predicting transient events differ fundamentally from those detecting sustained hypoxemia. Therefore, clear identification of the intended clinical goal is essential for adopting any published model.

A distinctive feature of this phenotype is the pathophysiological divergence between POH under general anesthesia and POH under procedural sedation. This divergence is reflected in model design. One study developed separate models for (1) induction-to-procedure end and (2) induction-to-endoscopic intubation initiation [59], illustrating that timeframe-specific prediction may be required even within a single surgical context. Encouragingly, one study demonstrated good external validation [66]. POH models may be generalizable when appropriately adapted to local patient populations, procedural characteristics, and monitoring practices.

### 4.4. PRF

Twelve studies (12/63, 19.0%) addressed PRF (Table 1 and Table 2 and Table S6) [24,39,67,68,69,,70,71,72,73,74,75,76], with outcomes defined using diverse criteria. This heterogeneity limits direct comparison of model performance across studies and complicates its clinical interpretation.

Standardized endpoints are essential for broader generalizability. The Standardized Endpoints in Perioperative Medicine (STEP) initiative provides a consensus-based framework underpinning quality metrics [77,78,79]. Importantly, the STEP initiative definition of PRF reflects contemporary clinical practice by incorporating two escalating respiratory support modalities, namely high-flow nasal cannula therapy and noninvasive positive-pressure ventilation, both of which are increasingly recognized as clinically meaningful indicators of postoperative respiratory compromise [80,81]. When clinicians apply predictive models based on composite PRF definitions without reporting individual component outcomes, they should carefully consider updated clinical definitions. Traditional definitions of PRF and ARDS may not uniformly capture high-flow nasal cannula and noninvasive ventilation use, potentially limiting clinical interpretability and generalizability of composite respiratory failure models [82,83,84]. Considering insufficient external validation (Table 1), these models are best used as foundational references for developing institution-specific prediction tools.

### 4.5. PE

Eleven studies (11/63, 17.5%) [13,24,54,85,86,87,88,89,90,91,92] addressed PE-inclusive VTE, and none of them modeled PE as a standalone outcome (Table 1 and Table 2 and Table S7). Models targeting PE are inherently distinct from those developed for the VTE composite. The incidence of PE is substantially lower than that of the VTE composite, leading to differences in event rates, calibration challenges, and predictive performance. PE and DVT also differ in clinical impact, therapeutic urgency, and prognosis, with PE generally associated with higher morbidity and mortality. The choice of outcome definition therefore has important implications for model development and clinical application; clinicians should not only consider the selected risk factors and modeling approach but also the intended clinical objective (risk stratification, preventive anticoagulation, or prognostic assessment) as these goals may require distinct endpoints and modeling strategies.

Only one study incorporated PE severity grading using the modified Clavien–Dindo classification [13]; all of the other models treated PE as a binary outcome. Severity grading reflects differences in physiological impact, therapeutic intervention, and prognosis, and models that do not account for PE severity may have limited clinical applicability; incorporating severity-aware endpoints can improve risk stratification and better align predictive models with clinically meaningful outcomes.

The International Classification of Diseases (ICD) codes facilitate large-scale model development; however, reliance on administrative data may underestimate PE incidence. Clinically significant PE can be missed when coding depends on symptom recognition, resulting in mild or asymptomatic cases going undetected. The observation window further influences outcome ascertainment. Several studies limited PE detection to initial hospitalization, yet DVT may develop during prolonged immobilization, and subsequent PE can occur after discharge, a limitation particularly relevant in healthcare systems with early transfer to rehabilitation or lower-acuity centers (frequently observed in the United States), potentially leading to missed post-discharge PE events.

### 4.6. Less-Studied PPRC Phenotypes

AI/ML evidence remains limited or absent for several PPRC phenotypes. For example, only one AI-based study (1/63, 1.6%) [93] modeled the risk of postoperative pulmonary edema (Table 1 and Table 2 and Table S8). Reliance on postoperative chest radiographs for outcome ascertainment introduced additional misclassification risk, as postoperative opacities can reflect atelectasis, pleural effusion, pneumonia, or fluid overload rather than true pulmonary edema [94,95,96]. Moreover, the model did not distinguish cardiogenic edema from non-cardiac edema, an etiologic distinction critical for treatment selection and prognosis. Pulmonary edema was less represented compared with other PPRC phenotypes within the scope of our search, potentially reflecting phenotype-specific complexity, diagnostic ambiguity across etiologic subtypes, a lack of standardized registry-compatible definitions, or limited prioritization within AI/ML research; however, this finding should not be interpreted as an exhaustive characterization of the available literature.

**Table 2:** Phenotype-specific clinical reference for clinicians across 63 study-listings: outcome definitions, surgical contexts, and clinical limitations of PPRC AI/ML models.

Phenotype (*n*)	Outcome Definition	Surgery/Procedure Types Reviewed	Key Clinical Limitations	Clinical Considerations for Local Implementation
Combined PPCs (*n* = 10)	Composite outcome incorporating multiple pulmonary events. Most common: EPCO consensus criteria, STEP-COMPAC composite endpoints, or study-specific composites combining pneumonia, respiratory failure, pleural effusion, atelectasis, and bronchospasm. Heterogeneity warning: composite definitions vary considerably across studies, with different component events and severity thresholds.	Lung/thoracic-dominant: Lung cancer surgery; Lung resection Thoracoscopic surgery (2 studies) Abdominal: Laparoscopic hepatectomy Emergency: Emergency gastrointestinal surgery Emergency cerebral hemorrhage surgery Mixed: General anesthesia; Mixed surgical population; Mixed non-cardiac under GA	(1) Outcome heterogeneity: composite definitions are not interchangeable across studies; the same model may classify different events as ‘PPRC’ depending on which consensus framework is used. (2) Limited external validation: only 1 of 10 studies (Peng, X. et al. 2022 [35]) conducted true cross-institutional external validation. (3) Some studies using prospective temporal validation showed changes in model performance, with the direction and magnitude of change varying across studies, underscoring that performance may differ according to the validation strategy and setting.	Most relevant to overall preoperative PPC risk stratification in surgical contexts represented in the available evidence. Before local implementation, institutions should verify that the composite outcome of the selected model corresponds to the clinical events relevant to their intended use, because differences in composite definitions may affect risk interpretation and subsequent clinical decision-making.
Pneumonia/ pulmonary infection (*n* = 21)	Postoperative pneumonia diagnosed using consensus or comparable criteria: CDC/NHSN, ATS, IDSA/ATS, EPCO, or Chinese HAP/VAP guidelines. Most studies require imaging confirmation (new or progressive infiltrate on chest imaging) plus at least one clinical sign (fever, leukocytosis, purulent secretions, or hypoxemia). Event rates: 1.54–62.3% across cohorts—a more than 40-fold variation. The lowest rate (1.54%) was reported in a broad surgical population (Xiang, B. et al. 2024 [53]); the highest rates were reported in liver transplantation (42.8%, Chen, C. et al. 2021 [40]) and critically ill post-neurosurgical patients (62.3%, Hu, A. et al. 2025 [46]), reflecting how patient selection dominates pneumonia incidence.	Most diverse surgical context coverage: Neurosurgical (5): Intracranial aneurysm embolization/clipping; neurosurgery; traumatic brain injury surgery; aneurysmal subarachnoid hemorrhage; acute spinal cord injury. Orthopedic (4): Hip fracture surgery (3 studies); lower limb fracture surgery. Cancer (3): Lung cancer high-risk surgery; hepatic carcinoma surgery; major surgery. Transplantation (2): Liver transplantation; deceased-donor kidney transplantation. Cardiac (1): Elective cardiac surgery. Other (6): Emergency general surgery; non-cardiac surgery; general surgical population; mixed adult surgical (2); mixed surgical (ACS-NSQIP).	(1) Geographic narrowness: Chinese single-center evidence dominates (Table S4); generalizability to other populations (different microbiology, antibiotic-use patterns, ICU practices) is uncertain. (2) Limited EV: only 3 of 21 studies conducted cross-institutional EV (Li, X. et al. 2024 [49], Dai, A. et al. 2024 [43], Bonde, A. eta la. 2021 [24]). (3) Overfitting risk: one single-center study reported an AUROC > 0.95 in a small cohort, but such high performance may not be maintained during EV.	Most clinically deployable phenotype with the broadest surgical coverage: hip fracture, neurosurgical, transplantation, and cancer surgery contexts all have dedicated published models. Critically, baseline pneumonia incidence varies more than 40-fold across published cohorts (1.54–62.3%), so a model’s calibration and decision thresholds are unlikely to transfer between low-incidence general surgery and high-incidence settings such as liver transplantation or post-neurosurgical ICU care. When selecting a model for your institution, prioritize one developed in a surgical context and population similar to yours, with imaging-confirmed outcome ascertainment. When selecting a model for local implementation, institutions should prioritize models developed in clinically comparable populations and assess compatibility of outcome ascertainment and baseline event incidence. Model-predicted risk may inform clinical assessment within established perioperative care pathways; however, the reviewed prediction studies do not establish that model-triggered interventions improve patient outcomes.
Hypoxemia (*n* = 8)	SpO_2_ below predefined threshold values, most commonly <90% or <95% (with <92% used less frequently), with definitions varying by perioperative timeframe (induction, intraoperative, post-extubation, or postoperative). Time-stratified definitions: some studies stratify hypoxemia by induction phase, late intraoperative phase, and postoperative phase—each predicted with separate models.	Procedural-sedation-dominant landscape (unique among PPCs): Endoscopic procedures (4): Colonoscopy; EGD; gastroscopy; ERCP minimally invasive surgery. (1): Laparoscopic, robot-assisted gynecologic, or urologic surgery. Cosmetic (1): Liposuction Pediatric (1): Mixed pediatric non-cardiac surgeries Mixed adult (1): Mixed surgical procedures.	(1) Definition heterogeneity: hypoxemia thresholds and duration criteria differ across studies, making direct comparison difficult. (2) Procedural-sedation context: endoscopy-derived models may not transfer to general anesthesia or major surgery, and vice versa. (3) Real-time deployment requirements: intraoperative prediction models require continuous EHR-monitor integration, which most institutions lack. (4) Single-cohort limitation: 5 of 8 studies are single-center, limiting inter-patient and inter-institutional generalizability.	Most useful as decision-support for procedural sedation (endoscopy, ERCP, gynecologic/urologic laparoscopy) and as intraoperative monitoring augmentation in higher-risk patients. Most relevant to procedural sedation and intraoperative settings represented in the available evidence. The intended prediction window should be matched to the clinical context, because endoscopy-derived models may not transfer to major surgery under general anesthesia and timeframe-specific models may not transfer across perioperative phases. Predicted risk may support clinical assessment and preparedness within established care pathways, but specific model-guided interventions have not been established by the reviewed studies. Be cautious that endoscopy-derived models should not be transferred to general anesthesia for major surgery, and timeframe-specific models do not transfer well across perioperative phases.
Respiratory failure (*n* = 12)	Highly heterogeneous: definitions include Berlin ARDS criteria (objective PaO_2_/FiO_2_ ratio threshold), STEP-COMPAC composite (reintubation + prolonged intubation ≥ 24 h + HFNC/BiPAP + ARDS), prolonged mechanical ventilation (varying 24 h–7 d thresholds), reintubation alone, or ICD-coded postoperative respiratory failure. Berlin ARDS involvement: 3 of 12 studies (33%): 2 used Berlin ARDS alone (Zhang, H. et al. 2024 [76], Wu, W. et al. 2025 [73]), 1 included it as a composite component (Xu, M. et al. 2025 [74]).	Cardiac (3): Cardiac surgery (2 adult studies); consecutive neonates undergoing index cardiac surgery (pediatric NEPHRON). Liver transplantation (1). Thoracic (1): Pulmonary lobectomy. Emergency (2): Emergency general surgery (2 studies) Broad/registry (5): Non-cardiac surgery; mixed surgical procedures (2); mixed surgical (ACS-NSQIP); anesthesia services for any non-labor surgery or procedure	(1) Definitional heterogeneity: the same patient may be classified as having or not having ‘respiratory failure’ depending on which study’s definition is applied. This is the most heterogeneously defined PPRC phenotype. (2) Physician-dependent ascertainment: reintubation timing, HFNC/BiPAP initiation, and prolonged ventilation thresholds reflect institutional and individual clinician judgment. (3) Pediatric prediction is substantially harder than adult prediction; the published pediatric cardiac surgery model (Blinder, J.-J. et al. 2024 [68]) may be too weak for clinical deployment.	Most directly relevant in cardiac surgery, liver transplantation, pulmonary lobectomy, and emergency general surgery contexts. For preoperative risk stratification, models with clearly defined and reproducible outcome ascertainment should be prioritized. Most relevant to the surgical populations represented in the available evidence. Institutions should ensure that the model’s respiratory-failure definition corresponds to their intended clinical endpoint and should not transfer adult models to pediatric populations without appropriate validation. Predicted risk may inform perioperative assessment and respiratory-support planning within established care pathways, but the reviewed studies do not demonstrate outcome benefit from specific model-triggered interventions. Do not transfer adult respiratory failure models to pediatric populations without revalidation.
Pulmonary embolism/venous thromboembolism (*n* = 11)	The 11 studies addressing pulmonary embolism/venous thromboembolism (PE/VTE) show substantial outcome heterogeneity, with no study modeling PE as a standalone outcome. Outcome configurations include VTE composite (DVT + PE merged) [85,86,89,92]; both DVT and PE reported separately within multi-endpoint or multilabel frameworks [13,24,54,88,90,91]; DVT-only via Doppler-based ascertainment despite the paper labeling the outcome as VTE [86]. Event rates vary substantially across configurations: VTE composite 0.046–5.1%; PE (within multi-endpoint models) 0.22–0.5%; DVT-only (systematic Doppler) 17.3%.	Bariatric (2): Bariatric surgery; Roux-en-Y gastric bypass. Orthopedic (3): Primary and revision total hip and knee arthroplasty; total hip arthroplasty; total hip and knee arthroplasty. Cancer/specialized (2): Lung cancer surgery; surgeries of major sellar region tumors. Broad/registry (4): Mixed surgical (ACS-NSQIP); mixed adult surgical population; mixed surgical procedures (2).	(1) Outcome configuration heterogeneity: No study modeled PE as a standalone outcome. The 11 studies span four configurations—VTE composite (*n* = 4), DVT and PE separately within multi-endpoint frameworks (*n* = 6), and DVT-only Doppler despite “VTE” labeling (*n* = 1), with event rates varying substantially (VTE composite 0.046–5.1%; PE 0.22–0.5%; DVT-only Doppler 17.3%). Models trained on one configuration should not be applied as if they predicted another, given differential implications for prophylaxis decisions and downstream clinical actions. (2) Rare event challenge: with event rates as low as 0.3–1% for pure PE, even good discriminators may have limited positive predictive value at clinically relevant thresholds. (3) Fragmented evidence base: 6 of 11 studies are single-center, only 1 is institution-led multicenter [91], and only 2 conducted true cross-institutional EV. (4) Temporal validation fragility: registry-based models can fail dramatically on temporal validation (e.g., LR AUROC of only 0.6 on ITV in ACS-NSQIP, Lex, J.-R. et al. 2024 [88]), suggesting non-stationary outcome distributions.	When selecting a model, institutions should verify the predicted endpoint (PE, DVT, or composite VTE) and ensure that it corresponds to the intended clinical use, because these outcome configurations are not interchangeable. Predicted risk may complement established VTE assessment and management pathways, but the reviewed studies do not establish that model-guided modification of surveillance or thromboprophylaxis improves patient outcomes. Given the low event rates and limited external validation, local validation remains particularly important. Standardized data structures and more reproducible outcome ascertainment, including approaches using unstructured clinical data, may facilitate future multicenter validation.
Less-studied (pulmonary edema) (*n* = 1)	Radiologist-confirmed pulmonary edema on postoperative chest X-ray. Defined as new alveolar or interstitial pulmonary edema findings adjudicated by radiologists. Event rate: 2.8%	Mixed surgical cohort from two Korean institutions; institution-led multicenter design with internal validation only. No dedicated prediction models exist for: Cardiac-surgery-related pulmonary edema Transfusion-related (TRALI/TACO) pulmonary edema Obstetric pulmonary edema despite their distinct pathophysiology and high clinical relevance.	(1) Single-study evidence: only one published model; no external replication exists, and the clinical takeaway is provisional. (2) Discrimination–utility gap: high AUROC may mask poor precision in low-prevalence settings—PR-AUC was only 0.44 despite AUROC of 0.91. (3) Pathophysiology heterogeneity: cardiogenic, hydrostatic, and TRALI-related pulmonary edema have very different risk factors that a single mixed-surgery model is unlikely to capture.	Given the single-study evidence base, the model should be considered supplementary risk information rather than established clinical decision support. Local validation is required before implementation, and the reviewed evidence does not establish specific model-guided interventions. This phenotype remains an important evidence gap warranting dedicated prospective studies.

Note: Clinical considerations in this table refer to potential uses of model-predicted risk within established standards of care and should not be interpreted as evidence that model-triggered interventions improve patient outcomes. The reviewed studies predominantly evaluated prediction performance rather than changes in clinical management or outcomes resulting from model-guided care. Specific clinical actions should be determined according to applicable guidelines, patient characteristics, and local institutional practice. Abbreviations: ACS-NSQIP = American College of Surgeons National Surgical Quality Improvement Program; AI/ML = artificial intelligence/machine learning; ARDS = acute respiratory distress syndrome; AUROC = area under the receiver operating characteristic curve; BiPAP = bilevel positive airway pressure; CT = computed tomography; EPCO = European perioperative clinical outcome; EV = external validation; HAP = hospital-acquired pneumonia; ICD = International Classification of Diseases; ICU = intensive care unit; ITV = internal temporal validation; LR = logistic regression; MBSAQIP = Metabolic and Bariatric Surgery Accreditation and Quality Improvement Program; OMOP CDM = Observational Medical Outcomes Partnership Common Data Model; PaO_2_/FiO_2_ = arterial partial pressure of oxygen to fraction of inspired oxygen ratio; PE = pulmonary embolism; PPRC = perioperative pulmonary and respiratory complication; PR-AUC = precision–recall area under the curve; SpO_2_ = peripheral oxygen saturation; STEP-COMPAC = Standardized Endpoints in Perioperative Medicine–Postoperative Pulmonary Complications; TACO = transfusion-associated circulatory overload; TRALI = transfusion-related acute lung injury; VAP = ventilator-associated pneumonia; VTE = venous thromboembolism.


**Clinical Takeaway** AI applications have been investigated across a broad range of perioperative pulmonary and respiratory complication phenotypes; however, the maturity and depth of evidence vary considerably across outcomes and among studies addressing the same phenotype. Although encouraging performance has been reported for several PPRCs, the evidence remains dominated by retrospective studies, with substantial heterogeneity in outcome definitions, patient populations, input data, modeling approaches, and validation strategies. Consequently, apparently similar performance across studies does not necessarily indicate comparable model validity or transferability. These recurring differences extend beyond individual phenotypes and highlight the need for cross-cutting evaluation of methodological quality, model evaluation, and clinical applicability.


## 5. Cross-Cutting Methodological Considerations

Across 59 unique publications contributing 63 study-listings, four methodological patterns recurred regardless of surgical context, algorithm choice, or geographical origin (the discrepancy between the numbers of unique publications and study-listings arises because three publications [24,39,54] addressed multiple PPRC outcomes from a single dataset). First, despite the substantial number of published models, cross-institutional external validation remains uncommon. Second, phenotype definitions varied widely, from internationally standardized consensus criteria to investigator-specific definitions adopted ad hoc. Third, sample sizes varied by four orders of magnitude, from approximately 200 patients in small single-center cohorts to several million procedures in nationwide registry-based studies. Fourth, model development methodologies were diverse, encompassing conventional machine learning algorithms, deep learning architectures, and additional approaches, including nomograms, natural language processing for outcome detection, and unsupervised techniques such as clustering for risk stratification. These patterns are critical considerations for clinicians evaluating whether an AI/ML model will perform as reported when applied in their clinical setting, and they motivate the cross-cutting analysis described in the subsequent sections.

### 5.1. Outcome Definition and Assessment

Across the 63 study-listings in this review, outcome ascertainment quality emerged as a fundamental yet under-recognized determinant of AI/ML model performance and clinical deployability. Of the 63 study-listings, 39 (61.9%) were classified as TIER A, 15 (23.8%) as TIER B, and 9 (14.3%) as TIER C (Table S9). All hypoxemia studies used objective measurement; postoperative respiratory failure had the highest ICD code usage (25%); and pneumonia/respiratory infection had the lowest TIER A proportion (8/21, 38.1%).

For postoperative pneumonia, 8 of the 17 study-listings used international standards or comparable criteria. Four additional studies addressed the broader category of postoperative pulmonary infection, which lacks an internationally recognized standardized definition and was therefore defined heterogeneously. By contrast, hypoxemia studies determined peripheral oxygen saturation thresholds at the discretion of individual investigators rather than from a harmonized standard. Among respiratory failure studies, those exclusively addressing ARDS (2 of 12 study-listings) applied the Berlin definition consistently; however, when other outcomes were included or were not ARDS, definitions varied substantially. ICD diagnostic codes were used in 6 of the 63 study-listings (9.5%), entered by clinicians after diagnosis based on imaging or clinical criteria.

This phenotype-dependent heterogeneity has direct implications for cross-study interpretation. AI/ML performance estimates for postoperative pneumonia can be meaningfully compared across studies using internationally standardized criteria, whereas hypoxemia thresholds, pulmonary infection definitions, and non-ARDS respiratory failure outcomes remain sufficiently variable to necessitate caution while interpreting aggregated estimates. Wider adoption of standardized PPRC-specific frameworks would substantially improve comparability of future AI/ML PPRC research.

### 5.2. Validation Insufficiency

Validation is essential to establish transportability of AI models [97]. Although many studies in this review reported some form of validation, this was predominantly internal (split-sample and cross-validation), which does not guarantee performance in new settings [98]. As most studies were conducted at single centers, external validation across institutions was often lacking, leaving uncertainty about whether these models would maintain discrimination and calibration across different patient case mixes, clinical workflows, and data structures.

Validation requirements should be matched to the intended deployment scope and clinical consequences of model use rather than defined by a universal minimum standard. For institution-specific implementation, retrospective internal validation followed by appropriately designed temporal/local validation may establish whether model performance is preserved in the intended local population, although these steps alone should not automatically justify bedside use when predictions will influence clinical decisions. In such settings, prospective silent validation can provide an intermediate step to assess performance, data integrity, and workflow feasibility without influencing patient care, followed, where appropriate, by prospective clinical deployment and clinical-impact evaluation. Cross-institutional external validation becomes particularly important when transportability beyond the local institution is intended.

Cross-institutional external validation remained uncommon, being reported in only 9 of 59 unique publications (15.3%), corresponding to 11 of 63 phenotype-specific study-listings (17.5%). Among these study-listings, paired internal and external AUROC values were available for most; however, the reported validation settings and outcome configurations varied substantially (Table S10). Changes in AUROC between internal and external validation were heterogeneous in magnitude and direction, with some models showing modest decreases and others showing similar or higher discrimination in external cohorts. Thus, the available evidence does not support a general conclusion regarding the magnitude of performance degradation during external validation. Rather, the more fundamental translation gap is the scarcity of cross-institutional external validation itself.

Temporal validation is also critical, considering that perioperative practices and patient populations evolve over time [99]. However, the adequacy of temporal validation should be judged by whether the validation period captures clinically relevant changes in patient case mix, clinical practice, data collection, and, where applicable, seasonal variation, rather than by a universal minimum time interval; excessively long intervals may also reduce relevance to the contemporary deployment environment.

The modeling approach should also reflect the outcome observation structure. When follow-up varies substantially or censoring and competing events affect outcome ascertainment, time-to-event or competing-risk approaches may be more appropriate than fixed-window binary prediction; however, changing the endpoint structure of an existing model in this way constitutes substantive model redevelopment and requires corresponding revalidation and governance rather than simple local recalibration.

### 5.3. Feature Set Heterogeneity

Features incorporated into AI/ML PPRC models varied substantially across the 63 study-listings, with heterogeneity arising from three principal sources: data source available to investigators, intended clinical purpose and prediction window, and temporal pathophysiology of the specific PPRC phenotype.

First, data source choice imposes intrinsic constraints on feature availability. Public registry–and large database–based studies are confined to structured variables collected by registries. Studies based on the ACS-NSQIP [24,88], Metabolic and Bariatric Surgery Accreditation and Quality Improvement Program [85,89], and the BioStudies repository [37,38] inherit standardized but limited variable sets. Institution-based studies enjoy comparatively greater feature flexibility but remain constrained by the retrospective designs dominating this literature, requiring already existing data in structured EHRs. With 61 of the 63 study-listings using retrospective designs, feature engineering opportunities are largely determined by historical documentation patterns rather than variables ideal for prediction.

Second, the intended clinical purpose and timing of model application influence feature selection. Preoperative risk stratification models appropriately restrict inputs to preoperative variables [88], whereas models designed for real-time intraoperative prediction [63] or integrated preoperative–intraoperative prediction [33] incorporate temporally appropriate features for each prediction window. Violation of this temporal logic introduces methodological risks, particularly data leakage.

Third, the temporal pathophysiology of each PPRC phenotype dictates clinically logical feature timings. Intraoperatively developing phenotypes can legitimately incorporate intraoperative physiological, surgical, and anesthetic variables. By contrast, postoperatively developing phenotypes are typically modeled with preoperative-only feature sets, with intraoperative features added selectively. For clinicians evaluating AI/ML PPRC tools, feature set appropriateness should be judged not by feature count but by whether each feature is causally upstream of the predicted outcome and within the workflow window of intended clinical use.

### 5.4. High Methodological Diversity

#### 5.4.1. Algorithm–Phenotype Matching

Approximately 80% of the 63 study-listings applied multiple algorithms and reported the best-performing model. The final selected models were predominated by tree-based ensembles (54.0%), followed by deep learning (19%) and logistic regression (LR)/linear models (17.5%). The distribution of algorithms varied substantially across phenotypes (Table S11). Combined PPCs showed the highest proportion of LR/linear models (30%), suggesting that complex algorithms do not necessarily outperform simpler models. Pneumonia showed the highest nomogram development rate (5/21, 23.8%).

Most final hypoxemia models were tree-based ensembles, although other approaches, such as support vector machine and LR, were also selected for specific prediction endpoints. The frequent use of tree-based methods in this phenotype may be consistent with their ability to accommodate nonlinear relationships and interactions in temporally dynamic or real-time prediction settings; however, the available evidence does not establish that these algorithms are intrinsically better suited for hypoxemia than LR or other approaches. The included studies substantially differed in sample size, event rate, feature availability, preprocessing, model tuning, class-imbalance handling, and validation strategy, precluding direct inference of phenotype-specific algorithm superiority.

Similarly, none of the selected respiratory failure models used LR or other linear algorithms, in contrast to their use in approximately 24–30% of models for other PPRC phenotypes. One possible explanation is the heterogeneity and partly clinician-dependent nature of respiratory failure definitions, including prolonged ventilation thresholds, reintubation decisions, and composite criteria, which may generate complex relationships potentially captured by nonlinear models. However, this interpretation remains a hypothesis and was not directly demonstrated by the reviewed studies.

PE/VTE showed the most diverse algorithmic landscape. Particularly for rare outcomes with substantial class imbalance, greater algorithmic complexity does not necessarily translate into better performance, generalizability, or clinical usefulness, and simpler models may achieve comparable or better generalization. Overall, the observed distribution of algorithm families across PPRC phenotypes should be interpreted as a descriptive pattern of the current literature rather than evidence of intrinsic phenotype–algorithm matching.

#### 5.4.2. Explainability and Transparency

AI models are often considered “black boxes” given the difficulty of interpreting the internal decision-making of complex algorithms such as deep neural networks [100]. Explainable AI addresses this by developing methods to make these systems more transparent [101]. Feature importance provides global-level interpretability across the entire dataset [102], while local techniques, such as Shapley additive explanations (SHAPs), provide feature-specific insights for individual predictions, which is particularly valuable in high-stakes clinical domains where users or auditors require justification for each decision [103]. Global and local explainable AI methods are valuable for translating PPRC models into clinical practice [104,105]. Integrating these insights can guide institution-specific development by highlighting clinically meaningful predictors, identifying robust surrogate variables, and informing feature engineering aligned with local workflows. As attribution methods are sensitive to feature correlations and dataset shifts, reporting global and local explanations alongside stability checks enhances interpretability and trustworthiness [106].

#### 5.4.3. Feature Selection and Clinical Feasibility

PPRC modeling using EHRs often involves high-dimensional predictors, with many studies including large feature sets without demonstrating dimensionality control or parsimony [13,24,88,89,91,92]. High-dimensional models increase overfitting risk, particularly when PPRC events are limited [107], and reduce transportability. From an implementation standpoint, models requiring numerous variables are less practical, as missing data and inter-institutional documentation variability make it unlikely that all inputs will be consistently available in real-time. Emphasizing clinically feasible feature sets, supported by regularization, stability assessment, and external or temporal validation, enhances generalizability and deployability [108]. Feature selection also enables parsimonious, clinically interpretable tools such as nomograms. Several studies in this review reported nomogram-based PPRC risk calculators [42,52,56,57,58,60,86]. ML-based feature selection followed by nomogram construction represents a hybrid approach bridging modern AI methodology with traditional clinical interpretability. It leverages ML strengths in high-dimensional data while retaining the clinician-friendly scoring of regression. This may be particularly suitable for institution-level implementation where clinician acceptance of black-box AI remains limited; however, simplification from a complex AI model to a nomogram should be accompanied by validation to ensure that discrimination and calibration are preserved.

### 5.5. Model Evaluation: Discrimination, Calibration, and Utility

In AI models, discrimination refers to the model’s ability to correctly rank outcomes; calibration ensures that predicted probabilities reflect observed real-world frequencies; and utility quantifies practical benefits in a specific context [109,110]. Discrimination is commonly assessed using AUROC or C-statistic [111], and most studies in this review reported AUROC as the primary performance metric (Table 1 and Tables S3–S8). However, AUROC alone is insufficient for PPRC prediction models because PPRC outcomes are typically rare, and class imbalance can obscure clinically important deficiencies [112]. AUROC also does not convey positive predictive value or performance at clinically relevant operating thresholds [113]. AUROC should therefore be complemented with metrics sensitive to rare events including area under the precision–recall curve (AUPRC), positive and negative predictive values, and threshold-based measures [114], alongside calibration metrics and clinical utility assessments through decision curve analysis [115,116,117]. Models reporting both are more clinically informative than those reporting discrimination alone: calibration ensures that predicted risk aligns with observed event rates, while clinical utility evaluates whether the model provides tangible benefits at meaningful decision thresholds.

Model-evaluation practices further demonstrated substantial gaps beyond discrimination (Table S12). Among the 59 unique publications, calibration plots or curves were reported in 26 (44.1%), whereas quantitative assessment using a calibration intercept/calibration-in-the-large or calibration slope was uncommon (4/59 [6.8%] for each); Brier scores were reported in 22 (37.3%). AUPRC was reported in 22 (37.3%), positive predictive value or precision in 37 (62.7%), and negative predictive value in 15 (25.4%). Only 14 publications (23.7%) performed decision curve analysis. Class imbalance was explicitly addressed in 30 (50.8%) using oversampling or synthetic sampling alone (20.3%), undersampling alone (6.8%), class weighting or cost-sensitive learning (10.2%), or hybrid over-/undersampling approaches (13.6%). Importantly, the timing of resampling relative to data splitting or cross-validation was not always sufficiently reported to exclude information leakage. None of the 59 publications prospectively evaluated the effects of model deployment on clinical decisions, care processes, or patient outcomes.
**Clinical Takeaway** Reported discrimination alone is insufficient to determine whether an AI model is clinically reliable or transferable. Differences in outcome definitions, patient populations, input features, and validation strategies can substantially affect model performance across institutions, while algorithm suitability may also depend on the target phenotype and local data structure. Importantly, our review identified substantial gaps in evaluation beyond discrimination: quantitative calibration assessment, prevalence-sensitive measures such as AUPRC, and decision curve analysis were inconsistently reported, and prospective evaluation of the clinical impact of model deployment was absent. Class imbalance was addressed in only approximately half of the included publications, and insufficient reporting of resampling procedures may leave information leakage difficult to exclude. Therefore, evaluation of PPRC models should combine discrimination with calibration and clinical utility, use metrics appropriate to outcome prevalence, ensure leakage-safe model development and validation, and consider explainability and feature feasibility when assessing transferability to a target clinical environment.

## 6. Practical Framework for Institution-Level Implementation

### 6.1. The Translation Gap: Why Current AI/ML PPRC Models Fail to Reach the Bedside

Despite the substantial body of evidence represented by the 63 study-listings analyzed in this review, routine clinical deployment of PPRC AI/ML models remains rare. Three interacting barriers explain this persistent translation gap.

First, irrespective of model performance, adoption is determined by usability and clinical convenience. Clinicians adopt tools that integrate seamlessly into bedside workflow. Models requiring numerous input variables are rarely used in routine practice. This is evidenced by the observation that LR-based nomograms remained competitive against complex methods even in large cohorts [42].

Second, performance evidence remains limited and often misleading. Only 9 of the 59 unique publications (15.3%) reported cross-institutional external validation. Two studies exhibited overfitting signatures with internal AUROC values >0.98 [45,87]. AUROC systematically overestimates clinical utility for rare outcomes [114]. Critically, the downstream impact on complications, length of stay, cost, or mortality is rarely evaluated.

Third, implementation feasibility is another barrier to the translation of AI PPRC models. Adoption is constrained by variable availability in target EHRs, real-time integration challenges, and cohort/system heterogeneity across institutions. These barriers interact and compound one another; therefore, bridging the translation gap requires coordinated efforts across all three dimensions [118].

Several established frameworks provide methodological and implementation guidance for clinical AI [20,21,22,119,120], but differ in scope and were not specifically designed for the heterogeneous perioperative pulmonary and respiratory context. Table S13 compares representative clinical AI frameworks with the PPRC-specific framework proposed in this review. Rather than replacing these established frameworks, our framework complements them by translating their broader principles into an institution-level pathway tailored to PPRCs, with particular emphasis on phenotype definition, local data availability and feature feasibility, adaptation to local populations and workflows, deployment, and continuous monitoring.

### 6.2. Six-Step Framework

The three dimensions of the translation gap described above map to six actionable steps for institution-level AI/ML PPRC implementation. The individual components of the proposed six-step framework are not intended as newly developed implementation concepts. Rather, the framework synthesizes established principles of model evaluation, local adaptation, validation, workflow integration, and post-deployment monitoring into a practical institution-level pathway tailored to PPRCs. Since resource requirements and clinical value may change during implementation, resource and economic feasibility should be reassessed throughout the six-step pathway rather than considered only during the initial institutional assessment. Figure 3 illustrates this framework as a cyclic process.

Step 1: ASSESS—institutional readiness and clinical need. First, clinical need: when alternative risk stratification tools exist, the proposed AI/ML model must demonstrate meaningful incremental value in patient outcomes, workflow, or cost-effectiveness; when no existing tool addresses this need, the potential for outcome or workflow improvement should be evaluated. Second, institutional readiness: data sufficiency must be evaluated quantitatively (cohort size) as well as qualitatively (predictor availability, outcome ascertainment quality, and real-time integration capability) [121,122,123]. Sustained operational capacity, including personnel, computational infrastructure, and ongoing performance monitoring, must also be evaluated, as models that cannot be operationally sustained will not achieve translation regardless of clinical benefit or technical validation [124,125]. As resource requirements and clinical value may change during adaptation, validation, deployment, and monitoring, their feasibility should be reassessed throughout the implementation pathway rather than only at the initial assessment.

Step 2: SELECT—parsimonious model appropriate to phenotype. Complex models can outperform simpler approaches but are often cumbersome when it comes to bedside implementation due to extensive variable requirements, computational demands, overfitting risk, and reliance on manual input. Seven of the 63 study-listings used ML-based variable selection followed by development of a nomogram. A simpler LR model may be more effective than complex deep learning architectures when bedside deployment is the goal. Ma J et al. [58] deliberately chose LR over extreme gradient boosting for nomogram visualization. The choice should balance predictive performance with deployability.

Step 3: ADAPT—local calibration and definition alignment. Benchmark the published model against institutional data, and map predictor variables to local EHR availability, substituting proxy variables when necessary. Apply institution-specific recalibration along a graded spectrum, from predictor weights or hyperparameter retuning through partial model revision to complete redevelopment. Although international consensus definitions provide standardization advantages, institutional needs or national insurance/reporting frameworks may require modified outcome targets, necessitating definition adaptation aligned with local clinical and administrative requirements.

Common data models may further facilitate cross-institutional translation by harmonizing predictor definitions, coding systems, outcome phenotypes, and temporal data structures [126]. The Observational Medical Outcomes Partnership Common Data Model (OMOP CDM) can support more reproducible phenotyping and multicenter or federated analyses by providing a shared representation of heterogeneous EHR data. This is also relevant to local adaptation, because independently reconstructing predictors and outcomes at each institution may itself introduce additional heterogeneity. Mapping clinically relevant variables and outcome definitions to a common data structure may therefore reduce site-specific variation and facilitate more reproducible local and multicenter validation.

Step 4: VALIDATE—tailored to the deployment scope. Validation should progress as appropriate from retrospective internal validation to temporal/local validation, prospective silent validation, prospective clinical deployment, and clinical-impact evaluation. Cross-institutional external validation is important when broader transportability is intended, whereas rigorous local and temporal validation may be more relevant for institution-specific deployment [127]. Retrospective cross-validation or temporal validation alone should not be assumed sufficient when predictions will directly influence patient care. Validation metrics should align with clinical purpose [112]: sensitivity and negative predictive value for screening; calibration and decision curve analysis for decision support; and AUPRC for rare-event outcomes. Defining clinical purpose first, then selecting validation metrics, ensures that validation effort directly translates to implementation value.

Step 5: DEPLOY—EHR-integrated and user-centered. Models should be integrated directly into the EHR so clinicians can access AI outputs at the point of care [128]. Medical-specialized platforms can package and connect AI applications to hospital clinical systems. The integrated tool must be user-friendly within clinical workflow; additional interface layers increase burden, leading to alert fatigue, workaround behaviors, and tool abandonment. Treating AI deployment as a change management initiative rather than a software release substantially increases adoption success [128,129].

Technical requirements for EHR integration also depend on the temporal characteristics of the model. Static models based on preoperative or other fixed variables differ substantially from dynamic models requiring continuous or near-real-time data, such as intraoperative hypoxemia prediction, which require timely data acquisition and management of missing, delayed, or failed data feeds. Interoperability standards such as Fast Healthcare Interoperability Resources may facilitate data exchange across systems [130]. Importantly, predictions should be delivered within an actionable clinical window, because even an accurate model may have limited utility if its output is delayed or creates excessive workflow burden.

Step 6: MONITOR—performance drift and outcome impact. Even well-performing models may experience performance drift after deployment due to shifts in patient characteristics, clinical practice patterns, outcome definitions, and the healthcare system [131,132]. Post-deployment monitoring should therefore assess not only discrimination but also calibration, clinically relevant error rates, and changes in predictor distributions, as appropriate, to the model and its intended use. When predefined, context-specific criteria indicate meaningful deterioration. Institutions should determine whether recalibration, model updating or re-estimation, retraining, or temporary withdrawal is required, followed by appropriate revalidation before continued clinical use. Such actions should be driven by evidence of meaningful change rather than by a fixed updating interval. Meaningful AI monitoring does not simply entail tracking discrimination metrics [133]; it must demonstrate its contribution to actual patient health outcomes, such as reductions in mortality, lower complication rates, and shorter intensive care unit stays. Model updates should be subject to version control and institutional governance, with responsibility for reviewing model failures and approving modification, redeployment, or withdrawal clearly defined. Regulatory requirements, human oversight, privacy, cybersecurity, and failure-management procedures should also be considered according to the model’s intended use and local context. Continuous post-deployment monitoring is therefore essential to maintain clinical utility, with iteration cycling back to Step 1 as new evidence emerges.

Importantly, these six steps should be viewed as a continuous learning cycle rather than a linear implementation pathway. Performance monitoring generates new institutional evidence that feeds back into model refinement and subsequent deployment.

Fairness should be assessed throughout the model lifecycle rather than only after deployment. Differences in demographic characteristics, disease prevalence, clinical practice, data availability, and healthcare infrastructure may result in differential model performance across populations and patient subgroups [134]. Therefore, local evaluation should, where clinically relevant and supported by adequate sample sizes, assess subgroup performance rather than relying solely on aggregate AUROC. Relevant measures may include subgroup-specific discrimination, calibration, false-positive rates, and false-negative rates, with the choice of metrics guided by the model’s intended clinical purpose and the consequences of prediction errors. Importantly, successful external validation at the population level should not itself be interpreted as evidence of fair or equitable performance, because clinically important performance disparities may remain within subgroups. Fairness should consequently be considered during model selection and adaptation, explicitly assessed during validation, and continuously reassessed after deployment as patient populations and clinical environments evolve [135].
**Clinical Takeaway** Integration of AI for PPRCs into perioperative practice should be approached as a locally adapted strategy rather than as a simple transfer of an existing model. Institutions should first define the clinical target and intended purpose and select evaluation metrics aligned with that purpose. They should then assess compatibility between the original development setting and the local environment, considering differences in patient populations and event incidence, healthcare systems, EHR structures, coding and documentation practices, clinical practices, and data availability and collection. These considerations are important given the geographic concentration of the current evidence base and the potential differences across healthcare systems. Local, site-specific, and temporal validation should be performed as appropriate, followed by necessary adaptation and integration into a user-friendly clinical workflow. As clinical environments evolve and model performance and clinical utility may change over time, continuous post-implementation monitoring is essential. These steps should operate as an interconnected and iterative process, allowing reassessment and adaptation as local conditions and clinical needs change.

## 7. Recommendations for Clinician Practice

Clinicians are the largest professional group that use AI/ML PPRC models in clinical practice; active engagement is essential at every stage from development to bedside deployment. Clinicians can play the following roles depending on the stage:

Step 1: Assess—clinicians define clinical need and institutional readiness.

Clinicians must determine which PPRCs are worth addressing. They must evaluate the model’s predictive performance or clinical value and also its effect on actual workflows. Additionally, clinicians must qualitatively assess the clinical reliability of field data, particularly regarding data quality. It is also important to determine if the clinical workforce and institutional capacity required for continuous operation and maintenance are sufficient.

Step 2: Select—clinicians assess real-world usability.

Clinicians, as end users, play a key role in evaluating the burden and feasibility of using candidate models in real-world clinical settings. High performance alone is insufficient to consider a model appropriate if obtaining input variables is cumbersome or the outputs do not aid in clinical decision-making. This clinician feedback can be reflected not only in model selection but also in subsequent model development and refinement.

Step 3: Adapt—clinicians ensure clinical validity of local adaptation.

Simple statistical recalibration alone is insufficient when adapting an existing model to a local/institution-specific setting. Clinicians must determine whether the target definition and input features align with the local patient population, clinical practice, and the original intended use. It is the clinician’s role to assess whether clinical meaning is maintained when feature substitution or target definition modification occurs.

Step 4: Validate—clinicians align validation with intended clinical use.

Which validation metrics are important depends on the clinical purpose. Therefore, clinicians must participate in the process of determining necessary evaluation indicators, such as calibration and clinical utility, to align with the actual intended use and target, rather than mechanically evaluating only discrimination.

Step 5: Deploy—clinicians determine whether the model actually fits clinical workflow.

In this stage, the role of the clinician is particularly important as they are the actual end users. EHR integration itself does not imply successful deployment; clinicians must evaluate whether the model provides understandable information at the appropriate time without disrupting the existing workflow and can be used for actual decision-making. Continued usability is also important here.

Step 6: Monitor—clinicians provide the feedback loop.

Model performance and utility may change after deployment as factors such as patient mix, clinical practice, outcome definition, and healthcare systems evolve. Clinicians are likely the first to experience how these changes manifest in the actual field. Therefore, clinicians play a continuous role in identifying signals of change, requesting reassessment or model modification, re-evaluating the modified model, and providing feedback.

## 8. Limitations and Conclusions

Our literature search was limited to PubMed and Google Scholar. Other databases such as Embase, Scopus, Web of Science, and Institute of Electrical and Electronics Engineers (IEEE) Xplore may have identified further publications, particularly those in engineering and cross-disciplinary venues not indexed in PubMed. Further, the restriction to English language publications may have excluded relevant studies. The first-200 cutoff for Google Scholar screening reflects the pragmatic limits of that platform’s retrieval reproducibility. Stage-by-stage record counts for the study-selection process were not systematically recorded during the search process, which limits complete reconstruction of the study-selection flow. Future systematic reviews of this evidence base should follow the Preferred Reporting Items for Systematic Reviews and Meta-Analyses (PRISMA) 2020 reporting standards, including real-time documentation of records identified, screened, excluded with reasons, and included.

As this was a narrative review, we did not perform a formal study-level risk-of-bias assessment. TIER is an author-developed, pragmatic framework for classifying outcome ascertainment quality and has not been independently validated. Although informed by outcome-related principles in TRIPOD + AI and PROBAST, some classification decisions may involve judgment, particularly when outcome definitions are incompletely reported. TIER assignments were initially performed by one reviewer and subsequently reviewed by a second reviewer, with disagreements resolved by consensus; therefore, inter-rater agreement was not assessed. Future studies should evaluate the reproducibility and validity of TIER using independent assessors and refine its operational criteria where necessary.

Finally, substantial heterogeneity in outcome definitions, patient populations, model-development methods, algorithms, and validation strategies limits direct comparisons across studies. As some publications contributed to more than one PPRC phenotype, 63 phenotype-specific study-listings were derived from 59 unique publications and should not be interpreted as 63 independent publications. The internal–external AUROC comparisons presented in this review are descriptive and exploratory and should not be interpreted as pooled meta-analytic estimates. Likewise, the heterogeneity of the included studies precludes conclusions regarding the comparative superiority of specific algorithm families.

This review synthesizes AI/ML-based models across diverse PPRC phenotypes. Substantial heterogeneity was observed in study design, diagnostic definitions, and observation windows, with geographic evidence concentrated primarily in China, the United States, and South Korea. Differences in patient populations, EHR structures, coding and documentation practices, and perioperative workflows may therefore limit the generalizability of published models and the implementation considerations synthesized in this review to other healthcare systems. Despite these limitations, many studies identified clinically meaningful predictors through explainable AI approaches and demonstrated acceptable model performance. These findings suggest that rather than deploying generalized “one-size-fits-all” models, institution-specific model development and local adaptation are more likely to achieve successful short-term clinical application.

For successful implementation, the prediction window, outcome definition, usability, and clinical workflow integration must be clearly delineated. As PPRC occurrence and diagnosis timing vary across phenotypes, applicable predictor windows and available data sources differ across models, which affects data collection, workflow integration, and outcome ascertainment.

## 9. Future Directions

Future research should prioritize generalizability and real-world utility by addressing the following key areas:

Standardization of diagnostic definitions, observation windows, and features.

Validation strategies appropriate to the intended clinical purpose and deployment setting.

Comprehensive evaluation including calibration and clinical utility through decision curve analysis using pre-specified thresholds linked to actionable clinical decisions.

Time-aware modeling to prevent data leakage and ensure real-time prediction for perioperative risk monitoring.

Feasible deployment through parsimonious feature sets compatible with routine data collection in the intended clinical setting.

Beyond incremental improvements to existing algorithms, emerging approaches such as foundation models and large language model-based pipelines can extend the scope and utility of AI for PPRCs by facilitating scalable phenotype labeling, feature extraction from unstructured data, and decision support for clinicians. The ultimate success of perioperative AI will be determined not by predictive accuracy alone, but by its ability to improve patient care through sustainable integration into everyday clinical practice.

NLP and emerging large language model approaches may further expand the use of unstructured perioperative data, including clinical notes and radiology reports, and potentially improve phenotype ascertainment where administrative codes are insufficient. However, LLM-based approaches introduce additional challenges, including non-deterministic outputs, hallucination, prompt sensitivity, privacy concerns, computational requirements, and model drift. These approaches therefore require rigorous validation and governance before clinical implementation. Importantly, such LLM-based applications represent future possibilities rather than evidence established by the studies included in this review.

## Figures and Tables

**Figure 1:**
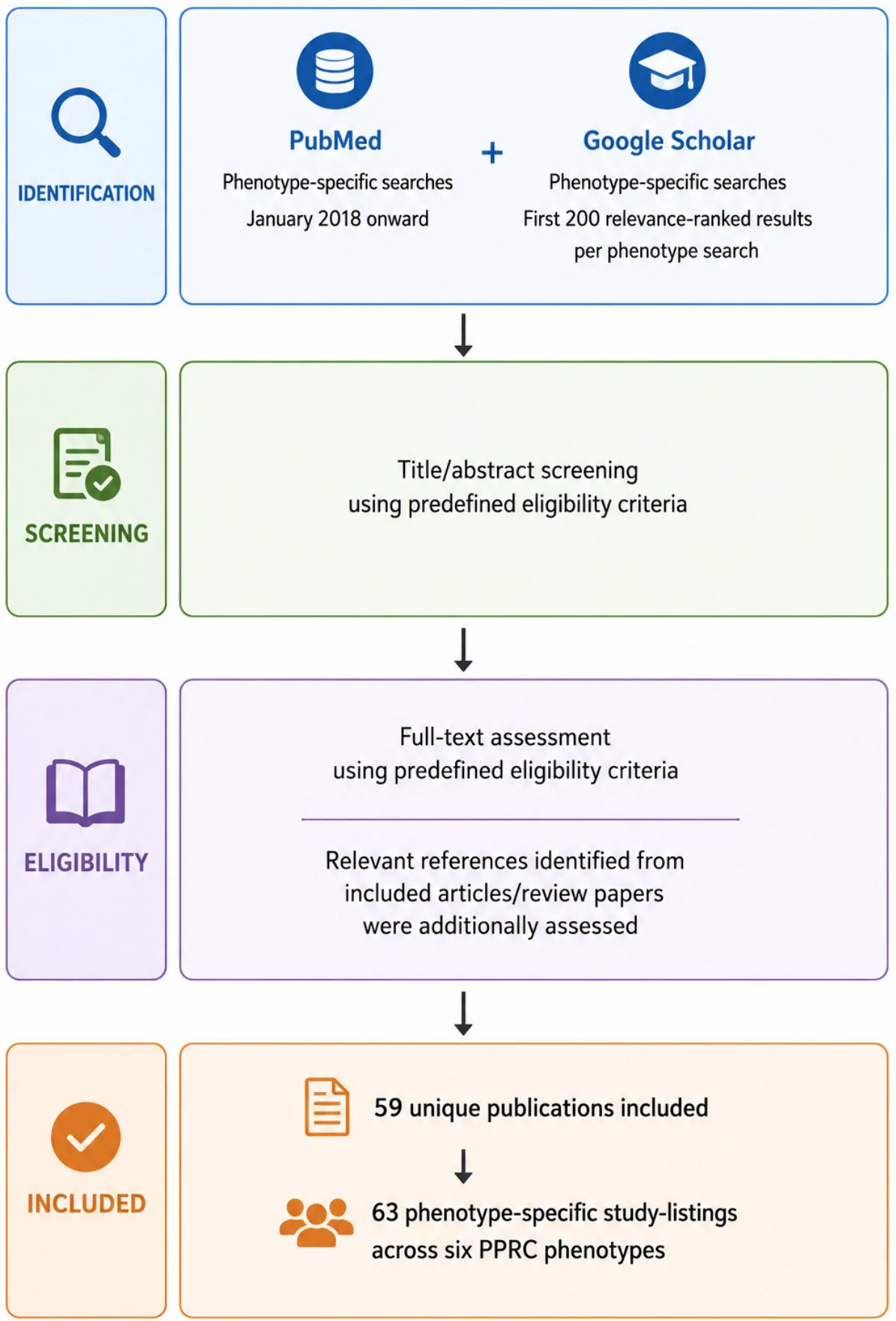
Literature search and study-selection process for the structured narrative review. PubMed and Google Scholar were searched using phenotype-specific strategies. For each Google Scholar search, the first 200 results ranked by relevance were screened. Records were assessed using predefined eligibility criteria, resulting in 59 unique publications contributing 63 phenotype-specific study-listings across six PPRC phenotypes. Stage-specific record counts were not prospectively documented and therefore not retrospectively reconstructed.

**Figure 2:**
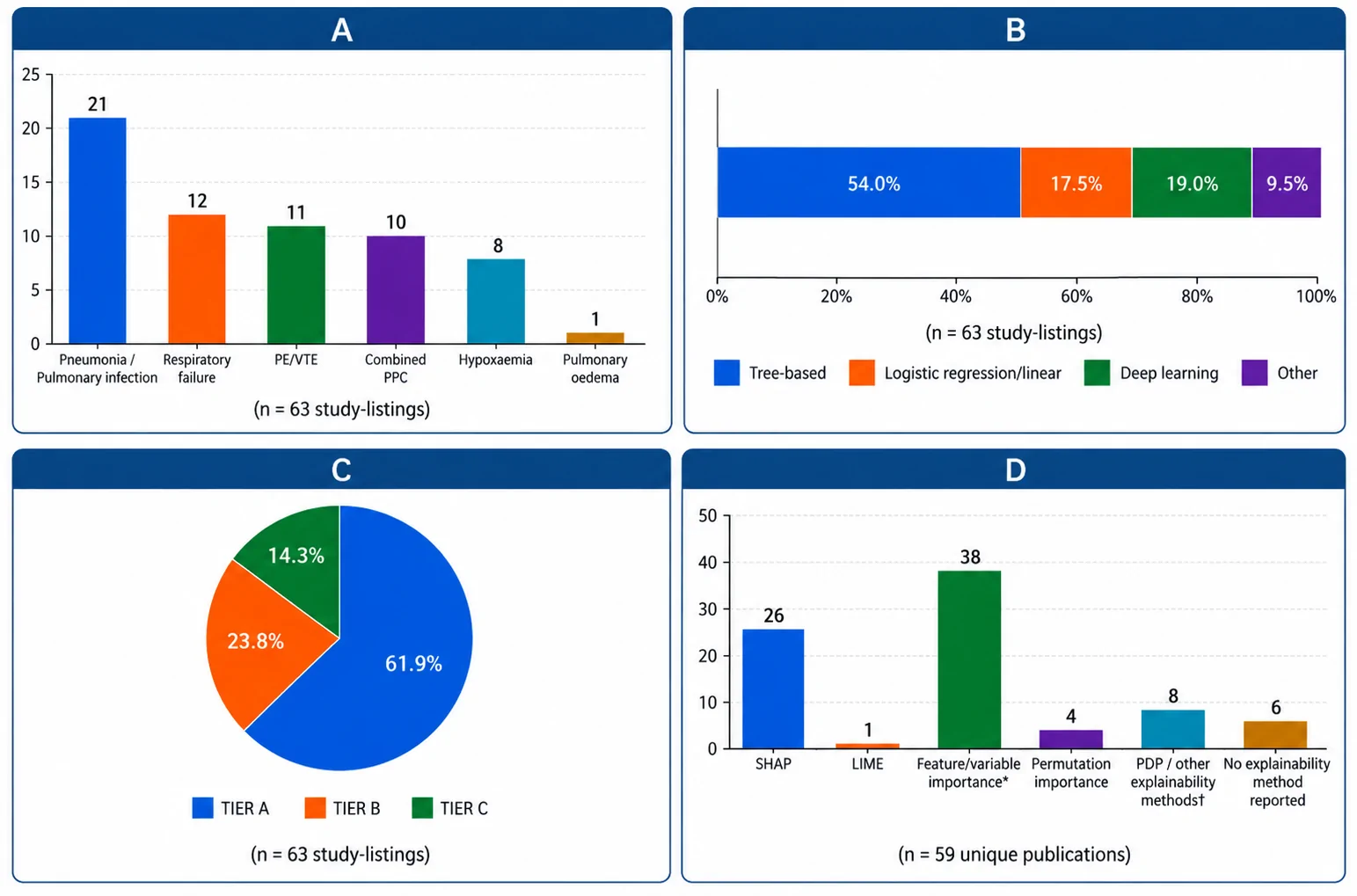
Overview of the artificial intelligence/machine learning landscape for perioperative pulmonary and respiratory complications. (**A**) Distribution of the 63 study-listings by phenotype. (**B**) Algorithm distribution across the 63 study-listings by phenotype (blue: tree-based, orange: logistic regression/linear, green: deep learning, purple: other). (**C**) Outcome ascertainment quality across the 63 study-listings (blue: TIER A, orange: TIER B, green: TIER C). (**D**) Explainable artificial intelligence method distribution across the 59 publications. Note: Explainability categories were not mutually exclusive; publications using multiple methods were counted in each applicable category. * Conventional feature/variable importance included model-native importance measures, regression coefficients or other variable-importance measures, mutual-information-based importance, and feature-importance rankings derived using SHAP. Accordingly, publications reporting SHAP-based feature importance could be counted in the SHAP and conventional feature/variable importance categories. † PDP/other explainability methods included partial dependence plots and other explicitly reported approaches not classified as SHAP, LIME, or permutation importance. SHAP-specific visualizations and analyses (e.g., dependence, waterfall/force, and interaction plots) were classified as SHAP and were not counted as PDP/other explainability methods. Abbreviations: LIME, local interpretable model-agnostic explanation; PDP, partial dependence plot; PE, pulmonary embolism; PPC, postoperative pulmonary complication; Resp, respiratory; SHAP, Shapley additive explanation; TIER, tiered framework for evaluating outcome ascertainment quality; VTE, venous thromboembolism.

**Figure 3:**
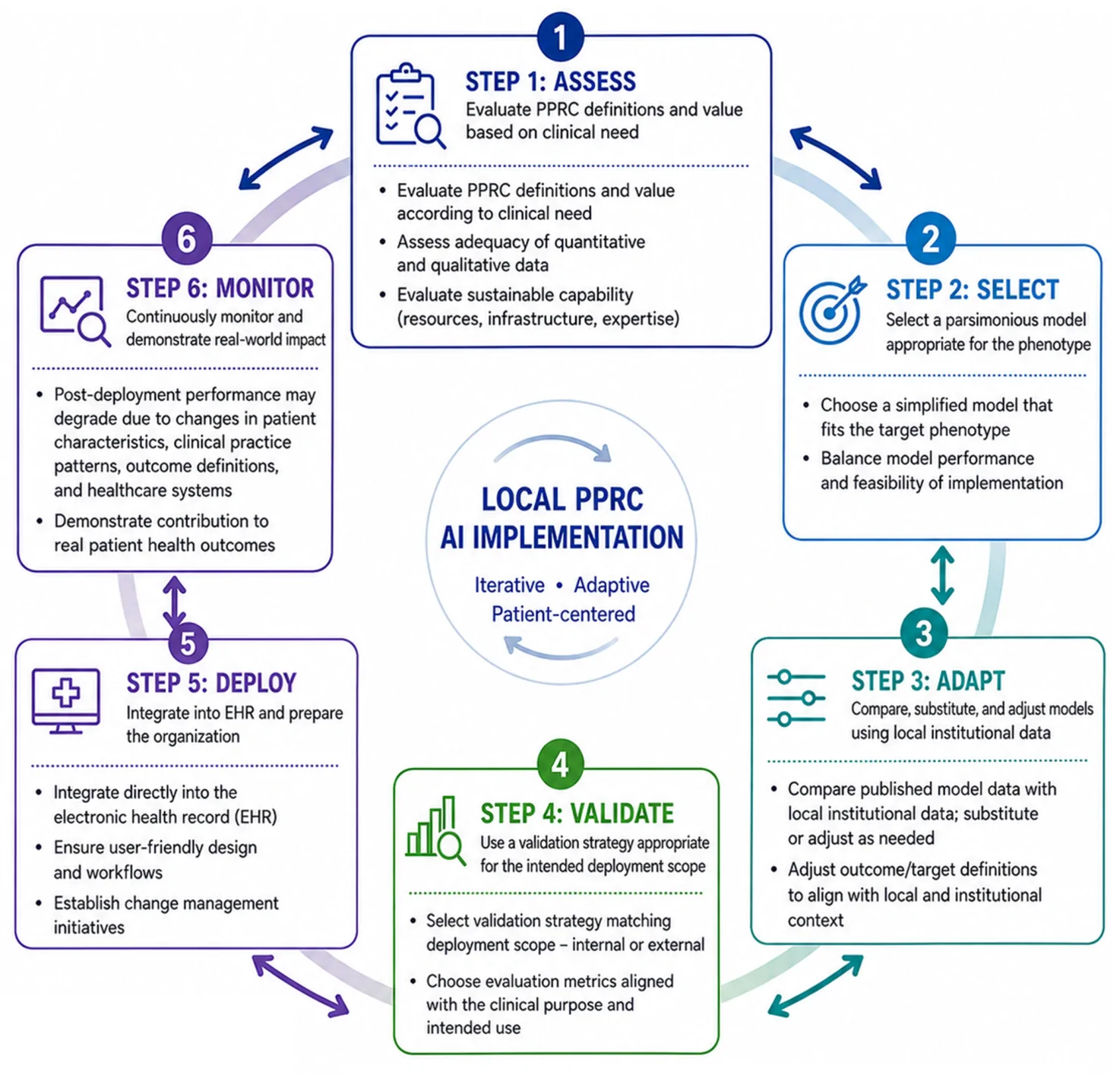
Six-step framework for institution-level AI/ML PPRC implementation. Fairness is a cross-cutting consideration throughout all six steps, from assessment and model selection to adaptation, validation, deployment, and post-deployment monitoring. Abbreviations: PPRC, perioperative pulmonary and respiratory complication.

## Data Availability

Data sharing is not applicable to this article as no datasets were generated or analyzed during the current study.

## Supplementary Materials

The following supporting information can be downloaded at https://www.mdpi.com/article/10.3390/diagnostics16193082/s1, Table S1: Detailed AI/ML definition and eligibility criteria; Table S2: Keyword combinations applied in the literature search for perioperative pulmonary and respiratory complications; Table S3: AI models of combined PPC across study-listings (10/63); Table S4: AI models of postoperative pneumonia/pulmonary infection across study-listings (21/63); Table S5: AI models of perioperative hypoxemia across study-listings (8/63); Table S6: AI models of postoperative respiratory failure across study-listings (12/63); Table S7: AI models of pulmonary embolism across study-listings (11/63); Table S8: AI models associated with less studied PPRC phenotypes (postoperative pulmonary edema) across study-listings (1/63); Table S9: TIER distribution across study-listings (*n* = 63) by phenotype; Table S10: Descriptive comparison of internal and external validation performance among PPRC AI/ML study-listings with cross-institutional external validation; Table S11: Distribution of algorithm by phenotype; Table S12: Reporting of model evaluation and clinical translation methods across included publications; Table S13: Comparison of established clinical AI methodological frameworks and the proposed PPRC-specific implementation framework.

## Abbreviations

The following abbreviations are used in this manuscript:

PPRC	Perioperative pulmonary and respiratory complication
PPC	Postoperative pulmonary complication
PE	Pulmonary embolism
VTE	Venous thromboembolism
DVT	Deep vein thrombosis
PRF	Postoperative respiratory failure
POH	Perioperative hypoxemia
ARDS	Acute respiratory distress syndrome
AI	Artificial intelligence
ML	Machine learning
NLP	Natural language processing
TIER	Tiered framework for evaluating outcome ascertainment quality
ACS-NSQIP	American College of Surgeons National Surgical Quality Improvement Program
STEP	Standardized Endpoints in Perioperative Medicine
AUROC	Area under the receiver operating characteristic curve
AUPRC	Area under the precision-recall curve
ICD	International Classification of Diseases
EHR	Electronic health record
TRIPOD	Transparent Reporting of a multivariable prediction model for Individual Prognosis or Diagnosis
PROBAST	Prediction model Risk-Of-Bias Assessment Tool
LR	Logistic regression
IEEE	Institute of Electrical and Electronics Engineers
PRISMA	Preferred Reporting Items for Systematic Reviews and Meta-Analyses
OMOP CDM	Observational Medical Outcomes Partnership Common Data Model
LLMs	Large language models
