# Supplementary material for: Bridging the Artificial Intelligence Translation Gap in Perioperative Pulmonary and Respiratory Complications: A Practical Framework for Institution-Level Implementation
Source: Diagnostics (Basel). 2026 Sep 23;16(19):3082. doi: 10.3390/diagnostics16193082 (PMC13664301; doi:10.3390/diagnostics16193082)
Supplement: Supplementary file 1 [file diagnostics-16-03082-s001.zip › diagnostics-4501000-supplementary.pdf]

Supplementary Information for Artificial intelligence for perioperative pulmonary complications: current evidence, translational barriers, and future directions

Table S1: Detailed AI/ML Definition and Eligibility Criteria

Domain	Detail
**AI/ML Definition**	
Classical supervised learning	Logistic regression, LASSO regression
Modern machine learning	Random forests, gradient boosting, support vector machines
Deep learning	Neural networks (DNN, CNN, RNN, Transformer)
Inclusion principle	Studies explicitly framed as machine learning or AI-based, regardless of specific algorithm
**Extended AI/ML Applications**	
Unsupervised learning	Clustering for patient stratification or phenotype identification
ML-assisted feature selection	LASSO or random forest importance followed by nomogram construction or hybrid statistical analysis
Rationale for inclusion	Reflects the full scope of AI/ML methodology in current PPRC research and provides complementary clinical utility beyond pure prediction
**Perioperative Care Definition**	
Surgical procedures	Under general, regional, or monitored anesthesia
Non-surgical procedures	Procedural sedation (gastrointestinal endoscopy, ERCP, aesthetic procedures)
Rationale	Reflects the breadth of clinical contexts in which perioperative pulmonary and respiratory complications (PPRCs) may occur
**Inclusion Criteria**	
(1) Methodology	AI/ML methodology addressing at least one PPRC outcome (prediction modeling, clustering-based stratification, or ML-assisted feature selection)
(2) Population	Perioperative patients (surgical procedures and procedural sedation); predominantly adults. Two pediatric studies were retained (one pediatric general anesthesia hypoxemia study and one neonatal cardiac surgery respiratory failure study) because of the scarcity of AI-based PPC evidence in pediatric populations.
(3) Quantitative outcomes	AUROC for prediction, statistical comparison metrics for clustering, or performance evaluation metrics for nomogram-based models
(4) Publication type	Original peer-reviewed articles
(5) Search & Publication Timeframe	Structured literature search of PubMed and Google Scholar conducted between January 2018 and November 2025 for English-language articles on AI/ML research for perioperative pulmonary and respiratory complications. Articles identified during this search window but with final publication dates after November 2025 (i.e., ahead-of-print or online-first articles at the time of search, such as Endo T. 2026 [47]) were retained in the review when their full text and final bibliographic information became available prior to manuscript finalization.
**Exclusion Criteria**	
Conference abstracts	Without full text
Editorials	All editorials
Study protocols	Without reported results
Off-topic articles	AI applications unrelated to perioperative pulmonary or respiratory outcomes

AI = Artificial Intelligence; AUPRC = Area Under the Precision-Recall Curve (equivalent to PR-AUC); AUROC = Area Under the Receiver Operating Characteristic curve; CNN = Convolutional Neural Network; DNN = Deep Neural Network; ERCP = Endoscopic Retrograde Cholangiopancreatography; GB = Gradient Boosting (umbrella term encompassing GBM, GBDT, XGBoost, LightGBM, CatBoost); LASSO = Least Absolute Shrinkage and Selection Operator; MAC = Monitored Anesthesia Care; ML = Machine Learning; PPC = Postoperative Pulmonary Complication; PPRC = Perioperative Pulmonary and Respiratory Complication (used in this review as the broader term encompassing both intraoperative and postoperative pulmonary and respiratory events; in this manuscript, "PPC" is used when referring specifically to postoperative outcomes following the established literature, while "PPRC" is used when referring inclusively to perioperative events including intraoperative hypoxemia); PROBAST = Prediction model Risk Of Bias ASsessment Tool (critical appraisal framework applied in this review); RF = Random Forest; RNN = Recurrent Neural Network; SVM = Support Vector Machine; TRIPOD+AI = Transparent Reporting of a multivariable prediction model for Individual Prognosis Or Diagnosis — extension for Artificial Intelligence (reporting guideline applied as the basis for critical appraisal in this review).

Table S2. Keyword Combinations Applied in the Literature Search for Perioperative Pulmonary and Respiratory Complications

	Google Scholar	PubMed
Combined PPCs	("machine learning" OR "deep learning" OR "neural network" OR "artificial intelligence"<br>OR "gradient boosting" OR "XGBoost" OR “random forest”)<br>AND<br>("postoperative" OR "perioperative" OR "surgical")<br>AND<br>("pulmonary complications" OR "pulmonary infection" OR "pulmonary edema" OR “atelectasis” OR “ pleural effusion”)<br>AND<br>("surgery")	("machine learning"[tiab] OR "deep learning"[tiab] OR "neural network"[tiab]<br>OR "artificial intelligence"[tiab] OR "gradient boosting"[tiab]<br>OR "xgboost"[tiab] OR "random forest"[tiab])<br>AND<br>("postoperative"[tiab] OR "perioperative"[tiab] OR "surgical"[tiab])<br>AND<br>("pulmonary complications"[tiab] OR "pulmonary infection"[tiab]<br>OR "pulmonary edema"[Mesh] OR "pulmonary edema"[tiab]<br>OR "atelectasis"[Mesh] OR "atelectasis"[tiab]<br>OR "pleural effusion"[Mesh] OR "pleural effusion"[tiab])<br>AND<br>("Surgical Procedures, Operative"[Mesh] OR "surgery"[tiab])<br>AND<br>("2018/01/01"[Date - Publication] : "3000"[Date - Publication])
Postoperative pneumonia and postoperative pulmonary infection	("postoperative pneumonia" OR "post-surgical pneumonia" OR "postoperative pulmonary infection" OR "post-surgical pulmonary infection") AND ("machine learning" OR "deep learning" OR "artificial intelligence") AND ("prediction" OR "risk model" OR "predictive model") AND (surgery OR perioperative OR intraoperative)	("postoperative pneumonia"[Title/Abstract]<br>OR "post-surgical pneumonia"[Title/Abstract]<br>OR "postoperative pulmonary infection"[Title/Abstract]<br>OR "post-surgical pulmonary infection"[Title/Abstract]<br>OR (pneumonia[MeSH Terms] AND postoperative[Title/Abstract]))<br>AND<br>("machine learning"[Title/Abstract]<br>OR "deep learning"[Title/Abstract]<br>OR "artificial intelligence"[Title/Abstract]<br>OR "machine learning"[MeSH Terms]<br>OR "artificial intelligence"[MeSH Terms])<br>AND<br>(prediction[Title/Abstract]<br>OR "risk model"[Title/Abstract]<br>OR "predictive model"[Title/Abstract]<br>OR "risk assessment"[MeSH Terms])<br>AND<br>(surgery[Title/Abstract]<br>OR surgical[Title/Abstract]<br>OR perioperative[Title/Abstract]<br>OR intraoperative[Title/Abstract]<br>OR "perioperative period"[MeSH Terms])<br>AND<br>("2018/01/01"[Date - Publication] : "3000"[Date - Publication])
Perioperative hypoxemia	("machine learning" OR "deep learning") AND (anesthesia OR perioperative OR postoperative) AND (hypoxia OR hypoxemia)	("machine learning"[Title/Abstract] OR "deep learning"[Title/Abstract]<br>OR "artificial intelligence"[Title/Abstract])<br>AND<br>("anesthesia"[Title/Abstract] OR "perioperative"[Title/Abstract]<br>OR "postoperative"[Title/Abstract])<br>AND<br>("hypoxia"[Title/Abstract] OR "hypoxemia"[Title/Abstract])<br>AND<br>("2018/01/01"[Date - Publication] : "3000"[Date - Publication])

Postoperative respiratory failure	("machine learning" OR "deep learning" OR "artificial intelligence" OR "neural network" OR "risk model") AND ("postoperative" OR "perioperative" OR "surgical") AND ("respiratory failure" OR "reintubation" OR "ARDS" OR "acute respiratory distress syndrome" OR "prolonged mechanical ventilation" OR "mechanical ventilation for more than 48 hours" OR "mechanical ventilation >48 hours" OR "prolonged MV") AND ("surgery")	("Machine Learning"[Mesh] OR "Deep Learning"[Mesh]<br>OR "Artificial Intelligence"[Mesh]<br>OR "machine learning"[tiab] OR "deep learning"[tiab]<br>OR "artificial intelligence"[tiab]<br>OR "neural network*"[tiab] OR "risk model*"[tiab])<br>AND<br>("Surgical Procedures, Operative"[Mesh]<br>OR postoperative[tiab] OR perioperative[tiab]<br>OR surgical[tiab] OR surgery[tiab])<br>AND<br>("Respiratory Insufficiency"[Mesh] OR "Respiration, Artificial"[Mesh]<br>OR "Respiratory Failure"[tiab] OR "ARDS"[tiab]<br>OR "acute respiratory distress syndrome"[tiab]<br>OR reintubation[tiab]<br>OR "prolonged mechanical ventilation"[tiab]<br>OR "mechanical ventilation for more than 48 hours"[tiab]<br>OR "mechanical ventilation >48 hours"[tiab]<br>OR "mechanical ventilation > 48 h"[tiab]<br>OR "prolonged MV"[tiab])<br>AND<br>("2018/01/01"[Date - Publication] : "3000"[Date - Publication])
Pulmonary embolism	("machine learning" OR "deep learning" OR "artificial intelligence") AND ("perioperative" OR "postoperative") AND ("pulmonary embolism" OR "PE") AND ("prediction" OR "risk model" OR "validation")	("Machine Learning"[Mesh] OR "Artificial Intelligence"[Mesh]<br>OR "Deep Learning"[Mesh]<br>OR machine learning[tiab] OR artificial intelligence[tiab]<br>OR deep learning[tiab])<br>AND<br>("Perioperative Period"[Mesh] OR perioperative[tiab] OR postoperative[tiab])<br>AND<br>("Pulmonary Embolism"[Mesh] OR pulmonary embolism[tiab] OR PE[tiab])<br>AND<br>(prediction[tiab] OR "risk model"[tiab] OR "risk prediction"[tiab]<br>OR validation[tiab])<br>AND<br>("2018/01/01"[dp] : "3000"[dp])
Postoperative pulmonary edema	("machine learning" OR "deep learning" OR "artificial intelligence") AND ("perioperative" OR "postoperative") AND ("pulmonary edema" OR "PE") AND ("prediction" OR "risk model" OR "validation")	("Machine Learning"[Mesh] OR "Artificial Intelligence"[Mesh]<br>OR "Deep Learning"[Mesh]<br>OR machine learning[tiab] OR artificial intelligence[tiab]<br>OR deep learning[tiab])<br>AND<br>("Perioperative Period"[Mesh] OR perioperative[tiab] OR postoperative[tiab])<br>AND<br>("Pulmonary Edema"[Mesh] OR pulmonary edema[tiab] OR pulmonary oedema[tiab])<br>AND<br>(prediction[tiab] OR "risk model"[tiab] OR "risk prediction"[tiab] OR validation[tiab])<br>AND<br>("2018/01/01"[dp] : "3000"[dp])

AI = Artificial Intelligence; ARDS = Acute Respiratory Distress Syndrome; DL = Deep Learning; [dp] = Date of Publication (PubMed field qualifier; equivalent to "Date - Publication"); MeSH = Medical Subject Headings (PubMed controlled vocabulary); ML = Machine Learning; MV = Mechanical Ventilation; PE = Pulmonary Embolism (in this review's nomenclature; not used as abbreviation for pulmonary edema); PPC = Postoperative Pulmonary Complication; PPRC = Perioperative Pulmonary and Respiratory Complication; [tiab] = Title/Abstract (PubMed field qualifier for free-text search of article titles and abstracts); [Title/Abstract] = equivalent to [tiab]; XGBoost = Extreme Gradient Boosting (specific gradient boosting algorithm).

Table S3. AI Models of Combined PPC across study-listings (10/63)

Author (Year) / Country or DB	Sample size (*n*)	Surgery / Procedure type	AI/ML methodology	TIER / Outcome definition / Event rate	Validation strategy	Best model & AUROC (or relevant metric)	Key limitation
Chen, S. *et al.* (2025) [26] / China	706 (derivation) / 177 (IV) / 308 (ITV)	Lung cancer surgery	LR, LGBM, BPNN, RF, GNB, KNN, DT, SVM, XGB, PLS-DA, GBDT, and Boruta	•TIER A<br>•StEP-COMPAC consensus; Atelectasis or pneumonia or ARDS or pulmonary aspiration within 7 days after surgery<br>•Event rate:<br>-Retrospective cohort: 35.4% (313/883)<br>-Prospective cohort: 29.5% (91/308)	Single center, IV + Prospective ITV	DT + SVM: 0.860 (IV), 0.790 (ITV)	•No EV<br>•Limited intraop. & anes. related data
Peng, X. *et al.* (2022) [27] / China	12,240 (derivation) / 7579 (EV)	General anesthesia	DNN, Compared with other models: Elastic Net logistic regression, SVM, RF, GBM, XGB	•TIER A<br>•EPCO criteria; Unplanned MV, atelectasis, pulmonary congestion, infection, effusion, pneumothorax, respiratory failure within 30 days after surgery<br>•Event rate:<br>-Derivation: 15.9% (1,949/12,240)<br>-External validation: 10.2% (776/7,579)	Multi center, EV	DNN: 0.884 (internal) / 0.889(external)	•Heavy model including 127 variables<br>•Limited intraop. & anes. related data<br>•Geographically limited EV<br>•Limited model interpretability
Xue, Q. *et al.* (2021) [24] / BioStudies database	926 (derivation 70%, validation 30%)	Emergency Gastrointestinal Surgery	LR, DT, GBDT, XBG, LGBM	•TIER C<br>•Atelectasis, pulmonary infections, pleural effusion, pulmonary thromboembolism<br>•A list of components is presented in other papers from the same group using the same cohort, but is not cited in this paper.<br>•Event rate: 20.19% (187/926)	Public repository, IV (five-fold cv)	GBM: 0.814	•No EV<br>•Limited sample size, and anes. related data<br>•Conceptual definition of outcome only<br>•Limited model interpretability
Qiu, X. *et al.* (2025) [22] / China	229 (derivation) / 57 (validation)	Thoracoscopic surgery	AutoML framework using IBSLO; LR, SVM, XGB, LGBM	•TIER C<br>•PPCs were defined as the occurrence of atelectasis, pleural effusion, persistent air leak, pneumothorax, or pneumonia. The authors stated that PPCs were assessed using	Single center, IV	AutoML-selected LGBM with IBSLO: 0.8775	•No EV<br>•Limited sample size, and anes. related data

				predefined criteria, but component-specific diagnostic criteria were not reported.<br>•Event rate: 89/286 (31.12%)			
Jing, X. *et al.* (2022) [19] / China	354 (derivation 70%, validation 30%)	Emergency cerebral hemorrhage surgery	KNN, SGD, SVC, RF, LR	•TIER B<br>•PPCs included pulmonary edema, pneumonia, pneumothorax, pulmonary embolism, or ARDS during hospitalization. Diagnoses were identified from critical care reports, radiographic reports, and/or discharge summaries with reference to previous studies; ARDS was explicitly diagnosed according to the 1994 American-European Consensus Conference criteria.<br>•Event rate: 190/286 (53.7%)	Single center, IV	LR: 0.6657	•No EV<br>•Low performance<br>•Limited sample size, and anes. related data<br>•Limited model interpretability
Li, S. *et al.* (2024) [21] / China	831 (derivation 70%, IV 30%) / 191 (ITV)	Laparoscopic hepatectomy	LR, SVM, LDA, RF, DT, AdaB, KNN	•TIER A<br>•StEP-COMPAC group consensus; atelectasis (radiological), pneumonia (CDC), ARDS (Berlin criteria); 7-day window<br>•Event rate: 8.7% (89/1,022, pneumonia 7.8%, atelectasis 0.6%, ARDS 1.8%)	Two center, IV + ITV	L2-regularized LR: 0.745 (IV), 0.680 (ITV)	•No EV<br>•Low performance<br>•Limited sample size
Zhou, C.-M. *et al.* (2024) [25] / BioStudies database	905 (derivation 70%, IV 30%)	Thoracoscopic surgery	LR, DT, RF, GB, XGB, LGBM, LSVC, MLP, GNB, KNN, AdaB, CNN, LSTM, CNNRNN, CNNLSTM, PBNN	•TIER A<br>•EPCO: Respiratory infection, respiratory failure, pleural effusion, atelectasis, pneumothorax, bronchospasm, aspiration pneumonia within 7 days after surgery<br>•Event rate: 22.9% (207/905)	Public repository, IV	PBNN: 0.869	•No EV<br>•Limited intraop. & anes. related data<br>•Limited model interpretability
Li, L. *et al.* (2024) [20] / China	180 (derivation 80%, validation 20%)	Lung resection	RF	•TIER A<br>•EPCO: Respiratory infection, respiratory failure, pleural effusion, atelectasis, pneumothorax, bronchospasm, aspiration pneumonia<br>•Event rate: 26.1% (47/180)	Single center, IV	RF: 0.86	•No EV<br>•Limited anes. related data<br>•Small sample size
Kim, J.-H. *et al.* (2023) [18]	111,212 (derivation 80%,	Mixed surgical population	LR, RF, MLP, XGB, LGBM	•TIER B<br>•Atelectasis (chest X-ray), pulmonary edema (chest X-ray),	Two centers, IV	Full-feature logistic	•No EV<br>•Heavy model

/ South Korea	validation20%)			pleural effusion (chest X-ray), pneumothorax (chest X-ray), pulmonary embolism (chest CT), respiratory failure (ventilator dependence ≥2 postoperative days), pneumonia (Pneumonia/infiltration + fever/WBC/blood culture), ARDS (Respiratory medical consultation)<br>•Event rate: 8.6% (9655/111,212)		regression: 0.767	including 127 variables<br>•Limited model interpretability
Li, P. *et al.* (2024) [23] / China	10,284 (derivation 80%, IV 20%) / 307 (ITV)	Mixed non-cardiac surgeries under general anesthesia	LR, Ridge regression, RF, GBDT, MLP, XGB	•TIER A<br>•EPCO: Mild/severe respiratory failure, ARDS, pulmonary infection, pleural effusion, atelectasis, pneumothorax, pulmonary edema within 7 days after surgery<br>•Event rate: Respiratory failure: 1.2% (125/10,484); ARDS: 0.0% (1/10,484); Respiratory infection: 8.2% (856/10,484); Pleural effusion: 11.0% (1148/10,484); Atelectasis: 1.4% (145/10,484); Pneumothorax: 5.6% (590/10,484); Pulmonary edema: 0.2% (18/10,484) in training set	Single center, IV (five-fold cv) + Prospective ITV	XGB: 0.878 (retrospective), 0.881 (prospective)	•No EV<br>•Limited anes. related data

Sample size is reported as Derivation cohort / Internal Validation (IV) / Internal Temporal Validation (ITV) / External Validation (EV) cohorts, where applicable. Percentages in parentheses indicate proportional split of total cohort when separate cohort sizes were not reported.

Validation strategy classification: Internal Validation (IV) = validation using held-out portion of derivation cohort via cross-validation or train-test split. Internal Temporal Validation (ITV) = validation using a temporally distinct cohort (chronologically later than derivation) within the same institution(s); prospective ITV represents a more rigorous internal validation strategy than retrospective cross-validation. External Validation (EV) = validation using an independent institutional dataset distinct from the derivation cohort.

Abbreviations (alphabetical):

AdaB = AdaBoost; anes. = anesthesia; ARDS = Acute Respiratory Distress Syndrome; AUROC = Area Under the Receiver Operating Characteristic curve; AutoML = Automated Machine Learning; Boruta = Boruta feature selection algorithm; BPNN = Back Propagation Neural Network; CDC = Centers for Disease Control and Prevention; CNN = Convolutional Neural Network; CNNLSTM = CNN combined with LSTM; CNNRNN = CNN combined with Recurrent Neural Network; cv = cross-validation; DB = Database; DNN = Deep Neural Network; DT = Decision Tree; EPCO = European Perioperative Clinical Outcome (consensus framework); EV = External Validation; GB = Gradient Boosting; GBDT = Gradient Boosting Decision Tree; GBM = Gradient Boosting Machine; GNB = Gaussian Naive Bayes; IBSLO = Iterative Boruta-SHAP-LASSO Optimization; intraop. = intraoperative; ITV = Internal Temporal Validation; IV = Internal Validation; KNN = K-Nearest Neighbors; LDA = Linear Discriminant Analysis; LGBM = Light Gradient Boosting Machine; LR = Logistic Regression; LSTM = Long Short-Term Memory; LSVC = Linear Support Vector Classifier; MLP = Multilayer Perceptron; MV = Mechanical Ventilation; PBNN = Probabilistic Bayesian Neural Network; PLS-DA = Partial Least Squares Discriminant Analysis; PPC = Postoperative Pulmonary Complication; RF = Random Forest; SGD = Stochastic Gradient Descent; StEP-COMPAC = Standardised Endpoints in Perioperative Medicine- Core Outcome Measures for Perioperative and Anaesthetic Care; SVC = Support Vector Classifier; SVM = Support Vector Machine; XGB = XGBoost.

Table S4. AI models of postoperative pneumonia/pulmonary infection across study-listings (21/63)

Author (Year) / Country or DB	Sample size (*n*)	Surgery / Procedure type	AI/ML methodology	TIER/ Outcome definition / Event rate	Validation strategy	Best model & AUROC (or relevant metric)	Key limitation
Xue, B. *et al.* (2021) [43] / USA	111,888 (derivation 80%, validation 20%)	Mixed adult surgical population	LR, SVM, RF, GBT, DNN	•TIER C<br>•ICD-10 diagnosis codes during hospitalization<br>•Event rate: 2.1%	Single center, IV (five-fold cv)	GBT: 0.905	•No EV<br>•ICD-10 diagnosis codes<br>•High-dimensional feature set limiting clinical practicality
Xiang, B. *et al.* (2024) [42] / China	396 (derivation) / 132 (validation)	General surgical population	KNN, SVM, RF, DT, GBM, AdaB, NB, GLM, LDA	•TIER A<br>•CDC criteria within 30 postoperative days<br>•Event rate:<br>-Source population incidence: 264/17,190 (1.54%)<br>-Modeling cohort event rate: 264/528 (50%, due to 1:1 matched design)	Single center, IV (ten-fold cv)	GLM & LDA: 0.877	•No EV<br>•Small sample size<br>•Limited model interpretability<br>•1:1 matched case-control design (model calibration may not transfer to unmatched source population where event rate is 1.54%)
Chen, C. *et al.* (2021) [29] / China	591 (derivation 80%, validation 20%)	Liver transplantation	LR, SVM, RF, GBM, XGB, AdaB	•TIER A<br>•EPCO definition based on radiologic and clinical criteria<br>•253/591 (42.8%)	Single center, IV (five-fold cv)	XGB: 0.734	•No EV<br>•Small sample size<br>•Limited metrics of liver donors<br>•Limited model interpretability
Chen, Y. *et al.* (2025) [30] / China	3539 (derivation) / 885 (validation)	Lower limb fracture surgery	XGB, SVM, KNN, RF, MLP, ET, AdaB, LGBM, TF	•TIER B<br>•American Thoracic Society healthcare-associated pneumonia guidelines<br>•228 / 3,539 (6.44%) in derivation cohort	Single center, IV (ten-fold cv)	Transformer: 0.866	•No EV<br>•Limited anes. related data
Chen, B. *et al.* (2024) [28] / China	7695 (derivation) / 3298 (validation)	Emergency general surgery	RF, SVM, NB, XGB, LR	•TIER B<br>•Diagnosed as such through imaging examinations<br>•Event rate: 5.75% (632/10,993)	Multi center, IV (ten-fold cv)	RF: 0.734	•No EV<br>•Low performance<br>•Limited anes. related data<br>•Limited model interpretability

Li, X. *et al.* (2024) [38] / China + MIMIC IV	565 (derivation) / 141 (IV) / 97 (EV)	Intracranial aneurysm embolization or aneurysm clipping	LR, SVM, RF, MLP, KNN, XGB	•TIER A<br>•CDC criteria within 30 postoperative days<br>•Event rate: 15.01% (training 16%, testing 12%)	Multi center IV + public database EV	LR: 0.91 (IV) / 0.89 (EV)	•Small sample size<br>•Limited anes. related data
Hu, A. *et al.* (2025) [35] / China	737	Neurosurgery	LR, SVM, CART, RF, AdaB, XGB, LGBM, CatB	•TIER B<br>•Chinese HAP/VAP guideline-based clinical and microbiological criteria<br>•Event rate: 459/737 (62.3%)	Single center, IV (ten-fold cv)	RF: 0.86	•No EV<br>•Small sample size<br>•Limited intraop. & anes. related data
Bonde, A. *et al.* (2021) [13] / ACS-NSQIP database	4,694,488 (training) / 1,173,622 (IV) / 13,771(EV)	Mixed surgical population (ACS NSQIP)	DNN	•TIER A<br>•ACS-NSQIP definition within 30 postoperative days<br>•Event rate: 0.2% (10582/4694467, in training); 2657/1 173 619 (0.2%, in validation); 15/13 771 (0.1%, in test)	Multicenter registry, IV + EV	Model 3(most complex DNN): 0.885 (IV) / 0.872 (EV)	•Limited anes. related data<br>•Fifty-eight input features
Cong, X. *et al.* (2025) [31] / China	31,320 (derivation) / 13,420(validation)	Non-cardiac surgery	LR, LASSO	•TIER B<br>•Radiologic and clinical criteria within 48 h after surgery<br>•Event rate: 3,187/44,740 (7.1%)	Single center, IV	LR: 0.804	•No EV<br>•Limited perioperative variable inclusion
Tang, M. *et al.* (2025) [40] / China	555 (derivation 70%, validation 30%)	Hip fracture surgery	RF, DT, XGB, SVM, RF, NN	•TIER B<br>•Radiologic, clinical, and microbiological criteria during postoperative hospitalization<br>•Event rate: 40/555 (7.2%)	Single center, IV	XGB: 0.929	•No EV<br>•Small sample size<br>•Limited anes. related data
Guo, J. *et al.* (2023) [34] / China	563 (derivation) / 242 (validation)	Hip fracture surgery	CART, GBM, KNN, LR, NN, RF, XGB	•TIER A<br>•Modified CDC criteria from 24 h after surgery to discharge<br>•Event rate: 75/805 (9.3%)	Single center, IV	XGB: 0.998	•No EV<br>•Small sample size<br>•Limited intraop. & anes. related data
Dai, A. *et al.*	498 (derivation 75%,	Hip fracture surgery	LR, RF, CatB, XGB, LGBM	•TIER B	Multi center,	DNN: 0.835	•Small sample size

(2024) [32] / China	IV 25%) / 124 (EV)			• ACS-NSQIP-based radiologic, clinical, and microbiological criteria<br>•Event rate:<br>-Derivation: 14.3%<br>-EV: 10.5%	IV (ten-fold cv) + EV	(internal) / 0.894 (external)	•Limited intraop. & anes. related data<br>•Geographically limited EV
Kim, J.-H. *et al.* (2023) [36] / South Korea	432 (derivation)/ 185 (validation)	Traumatic brain injury surgery	LR, BRF, LGBM, MLP, SVM, Hierarchical clustering	•TIER B<br>•Radiologic and laboratory criteria during hospitalization<br>•Event rate: 30.0% (185/617)	Multi center, IV	MLP + clustering: 0.8	•No EV<br>•Small sample size<br>•Limited model interpretability
Wang, T. *et al.* (2024) [41] / China	308 (derivation 70%, validation 30%)	Aneurysmal subarachnoid hemorrhage surgery	XGB, LR, RF, NB, KNN, SVM	•TIER A<br>•CDC criteria within 30 postoperative days<br>•Event rate: 23.4% (72/308)	Single center, IV	LR + Nomogram: 0.925	•No EV<br>•Small sample size<br>•Limited model interpretability<br>•Limited anes. related data
Lee, T. *et al.* (2023) [37] / South Korea	19,349 (derivation) / 4957 (validation)	Mixed adult surgical population	Deep learning–based computer-aided detection (DL-CAD)	•TIER A<br>•Antibiotic-treated respiratory infection with imaging-confirmed pulmonary infiltration within 30 postoperative days<br>•Event rate:<br>-Derivation: 1.0%<br>-Validation: 1.3%	Single center, IV	DL-CAD: 0.843	•No EV<br>•Limited anes. related data
Luo, Y. *et al.* (2020) [39] / China	519 (derivation 70%, validation 30%)	Deceased-donor kidney transplantation	RF, SVM, LR, NB, AdaB	•TIER A<br>•Modified IDSA/ATS hospital-acquired pneumonia guideline criteria during hospitalization<br>•Event rate: 43/519 (8.3%)	Single center, IV (ten-fold cv)	RF: 0.91	•No EV<br>•Small sample size<br>•Limited anes. related data<br>•Limited model interpretability
Endo, T. *et al.* (2026) [33] / USA	75 (derivation 70%, validation 30%)	Elective cardiac surgery	Neural network family (pcaNNet, avNNet, MLP with weight decay, NNet); KNN; RF; GLM; glmboost; NB; PAM;	•TIER B<br>•Clinical and radiologic criteria documented in medical records<br>•Event rate: 10/75 (13.3%)	Single center, IV	Diagnosis: RF: 0.833 / Prediction: GLBM: 0.833	•No EV<br>•Small sample size<br>•Limited clinical data<br>•Limited model interpretability

			SVM (linear, radial, radial-weighted)				
Ma, J. *et al.* (2025) [47] / China	1855 (derivation) / 795 (validation)	Lung cancer high-risk surgery	LR, ANN, SVM, RF, XGB	•TIER B<br>•Clinical, radiologic, and microbiological criteria within 1 week after surgery<br>•Event rate: 256/2,650 (9.66%)	Single center, IV (ten-fold cv)	XGB: 0.936	•No EV<br>•Limited anes. related data
Liu, J. *et al.* (2025) [45] / China	6637 (derivation) / 2844 (validation)	Major surgery	LR, DT, RF, SVM, GBDT, XGB, MLP	•TIER B<br>•Radiologic infiltration with clinical/laboratory criteria during hospitalization<br>•Event rate: 951/9,481 (10.03%)	Single center, IV (five-fold cv)	LR: 0.8	•No EV<br>•Limited clinical data
Li, M.-P. *et al.* (2023) [44] / China	610 (derivation) / 260 (validation)	Surgical treatment for acute spinal cord injury	RF, XGB, GBM, NBC, DT, LR	•TIER C<br>•Not reported<br>•Event rate: 98/870 (11.26%)	Single center, IV	RF: 0.721	•No EV<br>•Small sample size<br>•Low performance<br>•Limited anes. related data
Lu, C. *et al.* (2023) [46] / China	353 (derivation) / 152 (validation)	Hepatic carcinoma surgery	ANN, RF, GLRM	•TIER B<br>•Microbiological and clinical criteria during hospitalization<br>•Event rate: 86/505 (17.03%)	Single center, IV	RF: 0.801	•No EV<br>•Small sample size<br>•Limited anes. related data<br>•Limited model interpretability

Endpoint classification of included studies (21/63): Postoperative Pneumonia (n = 17): Xue, B. et al. (2021) [43] / USA; Xiang, B. et al. (2024) [42]/ China; Chen, C. et al. (2021) [29]/ China; Chen, Y. et al. (2025) [30]/ China; Chen, B. et al. (2024) [28]/ China; Li, X. et al. (2024) [38]/ China + MIMIC IV; Hu, A. et al. (2025) [35]/ China; Bonde, A. et al. (2021) [13]/ ACS-NSQIP database; Cong, X. et al. (2025) [31]/ China; Tang, M. et al. (2025) [40]/ China; Guo, J. et al. (2023) [34]/ China; Dai, A. et al. (2024) [32]/ China; Kim, J.-H. et al. (2023) [36]/ South Korea; Wang, T. et al. (2024) [41]/ China; Lee, T. et al. (2023) [37]/ South Korea; Luo, Y. et al. (2020) [39]/ China; Endo, T. et al. (2026) [33]/ USA.

Postoperative Pulmonary Infection (n = 4): Ma, J. et al. (2025) [47]/ China; Liu, J. et al. (2025) [45]/ China; Li, M.-P. et al. (2023) [44]/ China; Lu, C. et al. (2023) [46]/ China.

Sample size is reported as Derivation cohort / Internal Validation (IV) / Internal Temporal Validation (ITV) / External Validation (EV) cohorts, using the format reported in the original publication. When the original publication reported only proportional splits (percentages), the proportional split format is preserved.

Event rate is reported per cohort when separately reported in the original publication; otherwise, the overall event rate is provided. When event rate was reported only for the derivation cohort (e.g., Chen Y. 2025), the cohort-specific notation is preserved.

AI/ML methodology lists the algorithms evaluated in each study; the best-performing model with corresponding AUROC is reported in the "Best model & AUROC" column. When original publications used R caret package implementations with package-specific algorithm names (e.g., pcaNNet, avNNet, mlpWeightDecayML, glmboost, svmRadialWeights), these names are preserved for reproducibility.

Validation strategy classification: Internal Validation (IV) = validation using held-out portion of derivation cohort via cross-validation or train-test split. Internal Temporal Validation (ITV) = validation using a temporally distinct cohort (chronologically later than derivation) within the same institution(s); prospective ITV represents a more rigorous internal validation strategy than retrospective cross-validation. External Validation (EV) = validation using an independent institutional dataset distinct from the derivation cohort.

Abbreviations (alphabetical):

AdaB = AdaBoost; ACS-NSQIP = American College of Surgeons National Surgical Quality Improvement Program; anes. = anesthesia; ANN = Artificial Neural Network; ATS = American Thoracic Society; AUROC = Area Under the Receiver Operating Characteristic curve; avNNet = Model-Averaged Neural Network (caret implementation); BRF = Balanced Random Forest; CART = Classification And Regression Tree; CatB = CatBoost; CDC = Centers for Disease Control and Prevention; cv = cross-validation; DB = Database; DL-CAD = Deep Learning-based Computer-Aided Detection; DNN = Deep Neural Network; DT = Decision Tree; EPCO = European Perioperative Clinical Outcome (consensus framework); ET = Extra Trees; EV = External Validation; GBDT = Gradient Boosting Decision Tree; GBM = Gradient Boosting Machine; GBT = Gradient Boosted Trees; GLM = Generalized Linear Model; GLBM = Generalized Linear Boosted Model; glmboost = Boosted Generalized Linear Model (caret implementation); HAP = Hospital-Acquired Pneumonia; ICD-10 = International Classification of Diseases, 10th Revision; IDSA = Infectious Diseases Society of America; intraop. = intraoperative; ITV = Internal Temporal Validation; IV = Internal Validation; KNN = K-Nearest Neighbors; LASSO = Least Absolute Shrinkage and Selection Operator; LDA = Linear Discriminant Analysis; LGBM = Light Gradient Boosting Machine; LR = Logistic Regression; MIMIC IV = Medical Information Mart for Intensive Care IV; MLP = Multilayer Perceptron; NB = Naïve Bayes; NBC = Naive Bayes Classifier; NN = Neural Network (single-layer; corresponds to caret 'nnet'); NNet = Neural Network (caret implementation); NSQIP = National Surgical Quality Improvement Program; PAM = Prediction Analysis of Microarrays (Nearest Shrunken Centroids classifier); pcaNNet = Principal Component Step Neural Network (caret implementation); PPC = Postoperative Pulmonary Complication; PRAUC = Precision-Recall Area Under the Curve; RF = Random Forest; SMOTE = Synthetic Minority Oversampling Technique; SVM = Support Vector Machine; TF = Transformer; VAP = Ventilator-Associated Pneumonia; VOC = Volatile Organic Compound; XGB = XGBoost.

Table S5. AI Models of Perioperative hypoxemia across study-listings (8/63)

Author (Year) / Country or DB	Sample size (*n*)	Surgery / Procedure type	AI/ML methodology	TIER/ Outcome definition / Event rate	Validation strategy	Best model & AUROC (or relevant metric)	Key limitation
Lundberg, S.-M. *et al.* (2018) [55] / USA	42,420 (derivation) / 5649 (validation) / 5057 (test)	Mixed surgical procedures	XGB model named Prescience	•TIER A<br>•SpO_2 ≤92% during surgery under general anesthesia, MAC and regional anesthesia.<br>•Event rate: Not reported	Multicenter, IV (3-way split)	Prescience: 0.92	•No EV<br>•Limited clinical data
Lu, W. *et al.* (2024) [52] / China	671 (derivation) / 168 (IV)	Colonoscopy	LR, RF, XGB, SVM, stacking classifier	•TIER A<br>•Hypoxemia defined as SpO_2 <90% from anesthesia induction to completion of colonoscopy<br>•Event rate: 48/839 (5.7%)	Single center, IV	XGB: 0.913	•No EV<br>•Small sample size
Kim, J.-W. *et al.* (2025) [48] / South Korea	13,271 (derivation & validation) / 1289 (test)	Liposuction	XGB, RF, LR	•TIER A<br>•Respiratory depression defined as oxygen desaturation <95% requiring airway intervention or procedure cancellation<br>•Event rate:<br>-Train-validation: 637/13,271 (4.80%)<br>-Test: 62/1,289 (4.81%)	Single center, IV (3-way split)	XGB: 0.856	•No EV<br>•Limited anes. related data
Park, J.-B. *et al.* (2023) [49] / South Korea	1,273 (derivation) + 267 (internal validation) [= 1,540 development patients from 13,130 screened] / 200 (holdout	Mixed pediatric non-cardiac surgeries	GBM, LSTM, Transformer	•TIER A<br>•Intraoperative hypoxemia defined as SpO_2 <95% during general anesthesia<br>•Event rate:	Single center, IV (3-way split, five-fold cv)	GBM: 0.939	•No EV<br>•Limited anes. related data<br>•Limited model interpretability

	validation from 1,510 patients)			-11.7% (1,540/13,130 patients; 2,367 episodes)<br>-Holdout validation: 13.25% (200/1,510 patients; 289 episodes)			
Fang, Z. *et al.* (2023) [53] / China	928 (derivation) / 232 (validation)	Esophagogastroduodenoscopy	LR, SVM, RF, XGB	•TIER A<br>•Hypoxemia defined as SpO_2 <90% during gastroscopy without pulse oximetry artifact<br>•Event rate:<br>-Hypoxemia incidence 9.6% (112/1,160)<br>-Induction period 8.8% (102 cases)	Multicenter, IV + ITV + EV	Early intraoperative model (XGB): AUPRC, 0.377; Late intraoperative model (SVM): AUPRC, 0.541; Early induction model (XGB): AUPRC, 0.270; Late induction model (LR): AUPRC, 0.543	•External validation sample size and AUROC not reported<br>•Geographically limited external validation
Zheng, L. *et al.* (2025) [54] / China	688 (derivation) / 296 (IV) / 364 (EV)	Gastroscopy	LR, SVM, RF, XGB, LGBM	•TIER A<br>•Hypoxemia defined as SpO_2 <90% during the procedure<br>•Event rate: Internal cohort: 108/984 (11%); EV cohort event rate not separately reported	Multicenter, IV (ten-fold cv) + EV	RF: 0.914 (internal) / 0.848 (external)	•Geographically limited external validation<br>•Limited generalizability due to single-anesthetic protocol
Kang, H. *et al.* (2023) [50] / South Korea	4,280 (derivation) / 1,834 (validation)	Endoscopic Retrograde Cholangiopancreatography	LR, GBM ensemble (XGB)	•TIER A<br>•Hypoxemia defined as SpO_2 <90% during ERCP under monitored anesthesia care<br>•Event rate: 359/6,114 (5.9%)	Single center, IV (five-fold cv)	GBM ensemble model: 0.734	•No EV<br>•Limited anes. related data<br>•Low performance
Shin, Y. *et al.* (2023) [51] /	207 (derivation & IV) / 24 (ITV)	Laparoscopic, robot-assisted gynecologic, or urologic surgery	RF, XGB, LGBM, KNN,	•TIER A<br>•Post-anesthetic oxygen	Single center, IV (five-fold	RF: 0.812	•No EV<br>•Small sample size

South Korea			SVM	desaturation defined as recovery-room SpO_2 <95% despite oxygen supplementation<br>•Event rate: 74/207 (35.7%)	cv) + ITV		•Interpretability of derived spirometry features remains uncertain

Sample size is reported as Derivation cohort / Internal Validation (IV) / Internal Temporal Validation (ITV) / External Validation (EV) cohorts, using the format reported in the original publication. When the original publication reported only proportional splits (percentages), the proportional split format is preserved.

Event rate is reported per cohort when separately reported in the original publication; otherwise, the overall event rate is provided. For studies where multiple definitions or sub-cohort analyses are reported (e.g., Park J-B. 2023 with patient-level and episode-level reporting; Fang Z. 2023 [53] with time-stratified sub-models), the cohort-specific or sub-model–specific notation is preserved.

AI/ML methodology lists the algorithms evaluated in each study; the best-performing model with corresponding AUROC (or AUPRC where reported) is provided in the "Best model & AUROC" column. For time-stratified prediction approaches (Fang Z. 2023 [53]), separate best models are reported for each timeframe sub-model.

Validation strategy classification: Internal Validation (IV) = validation using held-out portion of derivation cohort via cross-validation or train-test split. Internal Temporal Validation (ITV) = validation using a temporally distinct cohort (chronologically later than derivation) within the same institution(s); prospective ITV represents a more rigorous internal validation strategy than retrospective cross-validation. External Validation (EV) = validation using an independent institutional dataset distinct from the derivation cohort.

Abbreviations (alphabetical):

anes. = anesthesia; AUPRC = Area Under the Precision-Recall Curve (equivalent to PRAUC); AUROC = Area Under the Receiver Operating Characteristic curve; cv = cross-validation; DB = Database; ERCP = Endoscopic Retrograde Cholangiopancreatography; EV = External Validation; GBM = Gradient Boosting Machine; intraop. = intraoperative; ITV = Internal Temporal Validation; IV = Internal Validation; KNN = K-Nearest Neighbors; LGBM = Light Gradient Boosting Machine; LR = Logistic Regression; LSTM = Long Short-Term Memory (recurrent neural network architecture); preop. = preoperative; Prescience = XGBoost-based real-time hypoxemia prediction model (Lundberg et al. 2018); RF = Random Forest; SpO_2 = Peripheral oxygen saturation; Stacking classifier = ensemble method that combines predictions from multiple base learners via a meta-learner; SVM = Support Vector Machine; Transformer = self-attention-based neural network architecture; XGB = XGBoost.

Table S6. AI Models of Postoperative Respiratory Failure across study-listings (12/63)

Author (Year) / Country or DB	Sample size (*n*)	Surgery / Procedure type	AI/ML methodology	TIER/ Outcome definition / Event rate	Validation strategy	Best model & AUROC (or relevant metric)	Key limitation
Xu, M. *et al.* (2025) [64] / China	711 (derivation) / 305 (validation)	Cardiac surgery	LR, MLP, XGB, CatB, DNN	•TIER A<br>•Composite postoperative respiratory failure including unplanned reintubation, prolonged ventilation >24 h, unplanned noninvasive respiratory support, or ARDS within 30 postoperative days<br>•Event rate: 16.3% (166/1,016)	Single center, IV (ten-fold cv)	DNN: 0.855	•No EV<br>•Limited cause-specific data<br>•Limited clinical integration
Kiyatkin, M.-E. *et al.* (2023) [62] / USA	59,388 (derivation) / 20,521 (validation) / 21,546 (test)	Anesthesia services for any surgery or procedure other than labor analgesia	RF	•TIER A<br>•Standardized Endpoints in Perioperative Medicine composite respiratory failure definition including reintubation, prolonged intubation ≥24 h, unplanned noninvasive respiratory support, or ARDS within 30 postoperative days<br>•Event rate: 1,813/101,455 (1.8%)	Single center, IV (3-way split)	RF: 0.93	•No EV<br>•Limited anes. related data<br>•Dependence on advanced EHR infrastructure<br>•Limited model interpretability
Zhang, H. *et al.* (2024) [66] / China	1,057 (derivation) / 436 (IV) / 503 (EV)	Cardiac surgery	DT, GBDT, AdaB, XGB, LGBM, RF, DF	•TIER A<br>•ARDS defined according to the 2012 Berlin criteria<br>•Event rate:<br>-Derivation: 11.5%; IV: 11.9%; EV: 15.1%	Multi center, IV + EV	DF: 0.886 / 0.827(I/E)	•Focused on ARDS<br>•Geographically limited external validation
Wu, W. *et al.* (2025) [63] / China	604 (derivation) / 151 (IV) / 115 (ITV)	Liver transplantation	LR, DT, RF, GBDT, NB, LGBM, XGB	•TIER A<br>•ARDS defined using Berlin criteria with PaO_2/FiO_2 ≤300 mmHg and bilateral pulmonary opacities within 7 days after surgery<br>•Event rate: derivation+IV: 30.99% (234/755); ITV: 24.35% (28/115)	Single center, IV + ITV	RF: 0.766 / 0.844 (I/T)	•No EV<br>•Small sample size<br>•Focused on ARDS

Yoon, H.-K. et al. (2024) [65] / South Korea	69,317 (derivation) / 29,708 (IV) / 60,923 (EV 1) / 139,864 (EV 2) / 7,521 (EV 3)	Non-cardiac surgery	GBM	•TIER A<br>•Postoperative mechanical ventilation >48 h or reintubation within 7 postoperative days<br>•Event rate:<br>-Derivation:0.42% (415/99,025)<br>-EV1: 0.22% (136/60,923)<br>-EV2: 1.00% (1,404/139,864)<br>-EV3: 0.49% (37/7,521)	Multi center, IV + 3-center EV	GBM: 0.912/ 0.872–0.931 (I/E)	•Geographically limited external validation
Hofer, I.-S. *et al.* (2020) [61] / USA	47,985 (derivation) / 11,996 (validation)	Mixed surgical procedures	DNN	•TIER A<br>•Postoperative reintubation before hospital discharge based on respiratory therapist documentation<br>•Event rate:<br>-Derivation: 548/47,985 (1.14%)<br>-IV: 159/11,996 (1.33%)	Single center, IV (five-fold cv)	DNN: 0.879	•No EV
Bonde, A. *et al.* (2021) [13] / ACS-NSQIP database (USA)	4,694,488 (derivation) / 1,173,622 (IV) / 13,771(EV)	Mixed surgical population (ACS NSQIP)	DNN	•TIER A<br>•Unplanned intubation requiring ventilator support or prolonged ventilation >48 h within 30 postoperative days<br>•Event rate:<br>-Unplanned intubation (derivation; IV; EV): 35,275 / 4,694,488 (0.8%); 8,815 / 1,173,622 (0.8%); 162 / 13,771 (1.2%)<br>-On ventilator for >48 h within 30 postoperative days (derivation; IV; EV): 35,265 / 4,694,488 (0.8%); 8,864 / 1,173,622 (0.8%); 149 / 13,7 71 (1.1%)	Multicenter registry, IV + EV	Unplanned intubation (DNN, Model 3): 0.914 (internal) / 0.902 (external); prolonged ventilation >48 (DNN, Model 3): 0.952 (internal) / 0.935 (external)	•Limited anes. related data<br>•Fifty-eight input features
Bihorac, A. *et al.* (2019) [57] / USA	41,166 (derivation) /	Mixed surgical procedures	MysurgeryRisk	•TIER C<br>• Respiratory failure defined using	Single center, IV (five-fold	RF: 0.82–0.94	•No EV<br>•Single-center model

	10,291 (validation)			ICD-9-CM codes<br>•Event rate: 14% (7,031/51,457)	cv)		with dependence on administrative coding and limited generalizability<br>•Limited model interpretability
Chen, B. *et al.* (2024) [28] / China	7695 (derivation) / 3298 (validation)	Emergency general surgery	RF, SVM, NB, XGB, LR	•TIER B<br>•Postoperative mechanical ventilation >48 h recorded in medical records<br>•Event rate: 6.13% (674/10,993)	Multi center, IV (ten-fold cv)	RF: 0.787	•No EV<br>•Limited anes. related data<br>•Potential bias from retrospective multicenter data and operator-dependent grading<br>•Limited model interpretability
Bolourani, S. *et al.* (2020) [59] / National Inpatient Sample (USA)	4062 (derivation 75%, validation 25%)	Pulmonary lobectomy	RF	•TIER C<br>•Respiratory failure defined using ICD-9-CM codes 518.81 and 518.82<br>•Event rate: 10.3% (417/4062)	Public database, IV	Corrected results after resolution of training–test data leakage: exact AUROC not reported (~10% reduction from originally reported values); sensitivity 40.1% and specificity 96.6% (RF alone); sensitivity 69.4% and specificity 85.0% (RF + SMOTEENN).	•No EV<br>•Potential coding bias and limited clinical detail in the administrative database<br>•Limited model interpretability
Blinder, J.-J. *et al.* (2024) [58] / Neonatal and Pediatric Heart and Renal Outcomes Network registry (USA)	782 (No separate validation cohort)	Consecutive neonates (age ≤ 30 days) undergoing index cardiac surgery	RF	•TIER B<br>•Prolonged postoperative mechanical ventilation/respiratory support ≥90th percentile of observed-to-expected ratio<br>•Event rate:<br>-Prolonged MV: 82/782 (10.5%)<br>-Prolonged RS: 80/782 (10.2%)	Multicenter registry analysis without separate validation cohort	Prolonged MV AUROC: 0.611 / Prolonged RS AUROC: 0.674	•No EV<br>•Low performance<br>•Retrospective multicenter design with unmeasured confounding and limited generalizability<br>•Limited model

							interpretability
Hadaya, J. *et al.* (2022) [60] / Nationwide Readmissions Database (USA)	1,003,703 (derivation 75%, validation 25%)	Emergency general surgery	XGB, LR	•TIER C<br>•Acute respiratory failure defined using ICD-10 diagnosis codes<br>•Event rate: 8.8%	Public database, IV	XGB: 0.9	•No EV<br>•Limited model interpretability<br>•Limited physiologic & anes. related data

Sample size is reported as Derivation cohort / Internal Validation (IV) / Internal Temporal Validation (ITV) / External Validation (EV) cohorts, using the format reported in the original publication. When the original publication reported only proportional splits (percentages), the proportional split format is preserved.

Event rate is reported per cohort when separately reported in the original publication; otherwise, the overall event rate is provided. For multi-outcome studies (e.g., Bonde A. 2021 [13] with both unplanned intubation and prolonged ventilation outcomes; Blinder J.J. 2024 [58] with both prolonged mechanical ventilation and prolonged respiratory support outcomes), separate event rates are documented per outcome.

AI/ML methodology lists the algorithms evaluated in each study; the best-performing model with corresponding AUROC (or relevant metric) is reported in the "Best model & AUROC" column. For studies using named composite models (e.g., MySurgeryRisk by Bihorac et al. 2019 [57]), the model name is preserved alongside its underlying algorithm.

Performance notation: "(I/E)" denotes Internal Validation / External Validation paired AUROC values; "(I/T)" denotes Internal Validation / Internal Temporal Validation paired AUROC values, using the convention reported in each original publication.

Validation strategy classification: Internal Validation (IV) = validation using held-out portion of derivation cohort via cross-validation or train-test split. Internal Temporal Validation (ITV) = validation using a temporally distinct cohort (chronologically later than derivation) within the same institution(s); prospective ITV represents a more rigorous internal validation strategy than retrospective cross-validation. External Validation (EV) = validation using an independent institutional dataset distinct from the derivation cohort.

Abbreviations (alphabetical):

AdaB = AdaBoost; ACS-NSQIP = American College of Surgeons National Surgical Quality Improvement Program; anes. = anesthesia; ARDS = Acute Respiratory Distress Syndrome; AUROC = Area Under the Receiver Operating Characteristic curve; CatB = CatBoost; cv = cross-validation; DB = Database; DF = Deep Forest (multi-layer ensemble of random forests); DNN = Deep Neural Network; DT = Decision Tree; EHR = Electronic Health Record; EV = External Validation; GBDT = Gradient Boosting Decision Tree; GBM = Gradient Boosting Machine; (I/E) = Internal Validation / External Validation paired AUROC; (I/T) = Internal Validation / Internal Temporal Validation paired AUROC; ICD-9-CM = International Classification of Diseases, 9th Revision, Clinical Modification; ICD-10 = International Classification of Diseases, 10th Revision; ITV = Internal Temporal Validation; IV = Internal Validation; LGBM = Light Gradient Boosting Machine; LR = Logistic Regression; MLP = Multilayer Perceptron; MV = Mechanical Ventilation; MySurgeryRisk = composite predictive analytics platform (Bihorac et al. 2019) using random forest as primary classifier; NB = Naïve Bayes; NEPHRON = Neonatal and Pediatric Heart and Renal Outcomes Network (pediatric cardiac surgery registry); NIS = National Inpatient Sample (USA public administrative database); NRD = Nationwide Readmissions Database (USA public administrative database); PaO_2/FiO_2 = Arterial partial pressure of oxygen to fraction of inspired oxygen ratio; PPC = Postoperative Pulmonary Complication; RESPIRE = Respiratory Failure Prediction model (Kiyatkin et al. 2023; random forest-based prediction tool); RF = Random Forest; RS = Respiratory Support; SMOTE = Synthetic Minority Oversampling Technique; StEP = Standardised Endpoints in Perioperative Medicine (consensus framework for outcome definitions); SVM = Support Vector Machine; XGB = XGBoost.

Table S7. AI Models of Pulmonary Embolism across study-listings (11/63)

Author (Year) / Country or DB	Sample size (*n*)	Surgery / Procedure type	AI/ML methodology	TIER/ Outcome definition / Event rate	Validation strategy	Best model & AUROC (or relevant metric)	Key limitation
Nudel, J. *et al.* (2021) [78] / MBSAQIP registry (USA)	218,403 (derivation) / 109,202 (validation) / 109,202 (test)	Bariatric surgery	ANN, XGB, LR	•TIER A<br>•Imaging-confirmed DVT or PE requiring anticoagulation, readmission, reintervention, reoperation, or death<br>•Event rate:<br>-VTE: 2,012/436,807 (0.046%)	Multicenter clinical registry, IV (3-way split)	XGB: 0.67	•No EV<br>•Limited anes. related data<br>•Limited model interpretability
Chen, Z. *et al.* (2025) [75] / China	710 (derivation) / 303 (test)	Lung cancer surgery	XGB, RF, DT, KNN, MLP, LR, SVM, NB	•TIER A<br>•Bilateral lower limb color Doppler ultrasonography before and after surgery to detect the presence or absence of deep vein thrombosis<br>•Event rate:<br>-VTE: 17.3% (175 / 1,013)	Single center, IV (ten-fold cv)	XGB: 0.66	•No EV<br>•Limited anes. related data<br>•Limited model interpretability<br>•Definition-actual ascertainment mismatch
Xue, B. *et al.* (2021) [43] / USA	111,888 (derivation 80%, validation 20%)	Mixed adult surgical population	LR, SVM, RF, XGB, DNN	•TIER C<br>•PE identified using ICD-10 diagnostic codes<br>•Event rate:<br>-PE: 0.5%	Single center, IV (five-fold cv)	XGB: 0.831	•No EV<br>•ICD-10 diagnosis codes<br>•High-dimensional feature set limiting clinical practicality
Shohat, N. *et al.* (2023) [81] / USA	35,963 (derivation: 70%; test: 30%)	Primary and revision total hip	RF, XGB, LASSO, SVM	•TIER A<br>•Symptomatic DVT or PE within 90	Single center, IV (five-fold cv)	GBT: 0.774	•No EV<br>•High model complexity

		and knee arthroplasty		postoperative days confirmed by imaging and treatment records<br>•Event rate:<br>VTE: 308/35,963 (0.86%) — includes 170 PE (0.47%) and 176 DVT (0.49%); 38 patients with both PE and DVT			limiting practical implementation<br>•Limited interpretability and prospective applicability of the ML model
Ding, R. *et al.* (2023) [76] / China	1036 (derivation) / 445 (test)	Total hip arthroplasty	LR, MLP, XGB, AdaB, GBC, KNN	•TIER C<br>•Discharge diagnosis of DVT or PE based on ICD-10 codes<br>•Event rate:<br>-VTE: 5.1% (76/1481)	Single center, IV (ten-fold cv)	XGB: 0.982	•No EV<br>•Limited anes. related data<br>•Limited postoperative follow-up and outcome granularity
Qiao, N. *et al.* (2023) [79] / China	2772 (derivation) / 1046 (validation)	Surgeries of major sellar region tumors	LR, penalized LR, SVM, LDA, RF, GBM, NN, Ensemble	•TIER A<br>•Ultrasound- or CT-confirmed DVT/PE within 60 postoperative days<br>•Event rate:<br>-60-day VTE incidence: 3.2% (124/3818)<br>-60-day PE (± DVT) incidence: 0.4% (16/3818)	Single center, IV (five-fold cv) + ITV	LDA: 0.869 (IV) / 0.884 (ITV)	•No EV<br>•Limited anes. related data<br>•Lack of potential underdetection of asymptomatic VTE
Ali, H. *et al.* (2024) [74] / MBSAQIP registry (USA)	6526	Roux-en-Y gastric bypass	Backward elimination LR	•TIER A<br>•DVT and/or PE within 30 postoperative days based on MBSAQIP criteria<br>•Event rate:<br>VTE: 0.84% (49/6,526)	Multicenter registry, IV (five-fold cv)	LR: 0.79	•No EV<br>•Limited anes. related data<br>•Lack of detailed center-level variables<br>•Limited interpretability
Selby, L.-V. *et al.* (2018) [10] / USA	DVT: 636 (derivation)/ 237 (validation); PE: 1285 (derivation) / 552 (validation)	Mixed surgical procedures	NLP-based (bag-of-words, WEKA	•TIER A<br>•NSQIP standardized definitions; NLP (bag-of-words classifier in WEKA) identifies DVT and PE separately from imaging reports (duplex ultrasound for DVT; CT angiography for PE)<br>•Event rate:<br>In validation set	Single center, IV	NLP (sensitivity [%] / specificity [%]): DVT, 85.1/94.6; PE, 90.0/98.7	•No EV<br>•Limited anes. related data<br>•Limited model interpretability<br>•Potential underdetection due to restricted imaging modalities and outcome

				-DVT: 6.7% (47/703)<br>-PE: 4.2% (30/703)			definitions
Shi, J. *et al.* (2021) [80] / USA	6735 (derivation) / 2266 (IV) / 16,493 (EV)	Mixed surgical procedures	NLP-based (EasyCIE rule-based)	•TIER A<br>•NSQIP definition for PE and DVT from the 2018 Operations Manual as the reference standard for the study; Both DVT and PE reported separately (NLP-based, separate metrics per endpoint)<br>•Event rate:<br>PE:<br>-Derivation: 0.22% (15/6735)<br>-IV: 0.31% (7/2266)<br>-EV: 65/16493 (0.39%)	Multicenter, ITV + EV	DVT detection: AUROC 0.920 (IV) / 0.921 (EV) vs ICD 0.761 (IV) / 0.794 (EV); p<0.001<br>PE detection: AUROC: 0.854 (IV) / 0.907 (EV); no significant difference vs ICD codes (NSS)	•Limited anes. related data<br>•Potential bias from retrospective multicenter data and operator-dependent grading<br>•Limited model interpretability
Lex, J.-R. *et al.* (2024) [77] / ACS-NSQIP database (USA)	394,724 (derivation) / 103,590 (validation)	Total hip and knee arthroplasty	LR, RF, XGB	•TIER A<br>• NSQIP-defined symptomatic DVT or PE identified from radiology reports within 30 postoperative days; three outcomes reported (DVT, PE, any VTE event)<br>•Event rate:<br>-VTE: 0.8% (4405/498,314)<br>-PE: 0.38% (1913/498,314)	Multicenter registry, IV (five-fold cv) + ITV	LR: 0.6 (ITV)	•No EV<br>• Potential bias from retrospective multicenter data and operator-dependent grading<br>•Limited model interpretability<br>•Low performance
Bonde, A. *et al.* (2021) [13] / ACS-NSQIP database (USA)	4,694,488 (derivation) / 1,173,622 (IV) / 13,771 (EV)	Mixed surgical population (ACS NSQIP)	DNN	•TIER A<br>•PE defined according to ACS NSQIP criteria within 30 postoperative days<br>•Event rate:<br>PE:<br>-Derivation: 15,379 / 4,694,488 (0.3%)<br>-IV: 3817 / 1,173,622 (0.3%)<br>-EV: 92 / 13,771 (0.7%)	Multicenter registry, IV + EV	Model 3 (most complex DNN): 0.813 (IV) / 0.763 (EV)	•Limited anes. related data<br>•Fifty-eight input features

Sample size is reported as Derivation cohort / Internal Validation (IV) / Internal Temporal Validation (ITV) / External Validation (EV) cohorts, using the format reported in the original publication. For studies reporting separate cohorts by imaging modality (e.g., Selby L.V. 2018 [10] with separate ultrasound and CT angiogram cohorts), the modality-specific notation is preserved. When the original publication reported

only proportional splits (percentages), the proportional split format is preserved.

Event rate is reported per cohort when separately reported in the original publication; otherwise, the overall event rate is provided. For multi-outcome studies reporting separate VTE and PE incidences (Qiao N. 2023 [88], Selby L.V. 2018 [10], Lex J.R. 2024 [77]), the cohort-specific notation is preserved. For studies reporting composite VTE outcomes with internal PE+DVT breakdown (Shohat N. 2023 [81]), patient overlap between PE and DVT events is noted in the event rate field (e.g., 170 PE + 176 DVT components with 38 patients having both events).

Observation periods: 30 days (n=6) [10, 13, 74, 77, 78, 80]; 60 days (n=1) [79]; 90 days (n=1) [81]; hospitalization-window or discharge-based (n=4) [43, 75, 76].

AI/ML methodology lists the algorithms evaluated in each study; the best-performing model with corresponding AUROC (or sensitivity/specificity for NLP-only studies without AUROC reporting, such as Selby L.V. 2018 [10]) is provided in the "Best model & AUROC" column. For studies using composite or pipeline approaches (e.g., Ali H. 2024 [74] with backward elimination multivariate logistic regression; Qiao N. 2023 [88] with ensemble), the methodological approach is preserved.

Performance notation: "(I/E)" denotes Internal Validation / External Validation paired AUROC values; "(I/T)" or "(IV/TV)" denotes Internal Validation / Internal Temporal Validation (Temporal Validation) paired AUROC values, using the convention reported in each original publication.

Validation strategy classification:

Internal Validation (IV) = validation using held-out portion of derivation cohort via cross-validation or train-test split.

Internal Temporal Validation (ITV) or Temporal Validation (TV) = validation using a temporally distinct cohort (chronologically later than derivation) within the same institution(s) or registry; prospective ITV represents a more rigorous internal validation strategy than retrospective cross-validation. Lex J.R. 2024 [77] used 2014–2018 procedures for training and validation, with 2019 TJAs for testing — an ITV design without separate institutional external validation.

External Validation (EV) = validation using an independent institutional dataset distinct from the derivation cohort. Among the 11 PE-VTE studies, only 2 conducted true cross-institutional EV: Bonde A. 2021 [13] (ACS-NSQIP 5.88M derivation with cross-institutional EV cohort of 13,771 patients) and Shi J. 2021 [80] (EasyCIE-PEDVT with internal and external validation cohorts). Registry-based design provides multi-institutional data sources but does not by itself constitute true cross-institutional external validation, which requires validation on a dataset independent of the derivation cohort.

Abbreviations (alphabetical):

AdaB = AdaBoost; ACS-NSQIP = American College of Surgeons National Surgical Quality Improvement Program; anes. = anesthesia; ANN = Artificial Neural Network; AUROC = Area Under the Receiver Operating Characteristic curve; CT = Computed Tomography; cv = cross-validation; DB = Database; DNN = Deep Neural Network; DT = Decision Tree; DVT = Deep Vein Thrombosis; EasyCIE = Easy Clinical Information Extractor (rule-based NLP tool; Shi J. 2021); EasyCIE-PEDVT = EasyCIE configured for PE and DVT detection; EV = External Validation; GBC = Gradient Boosting Classifier (scikit-learn implementation); GBT = Gradient Boosting Tree; GBM = Gradient Boosting Machine; ICD-10 = International Classification of Diseases, 10th Revision; (I/E) = Internal Validation / External Validation paired AUROC; (I/T) or (IV/TV) = Internal Validation / Internal Temporal Validation (Temporal Validation) paired AUROC; ITV = Internal Temporal Validation; IV = Internal Validation; KNN = K-Nearest Neighbors; LASSO = Least Absolute Shrinkage and Selection Operator; LDA = Linear Discriminant Analysis; LGBM = Light Gradient Boosting Machine; LR = Logistic Regression; MBE = Major Bleeding Event (separate outcome reported in Shohat N. 2023; not within the PE-VTE phenotype scope of this review); MBSAQIP = Metabolic and Bariatric Surgery Accreditation and Quality Improvement Program; MLP = Multilayer Perceptron; NB = Naïve Bayes; NLP = Natural Language Processing; NN = Neural Network; NSQIP = National Surgical Quality Improvement Program; NSS = Not Statistically Significant; PE = Pulmonary Embolism; PPC = Postoperative Pulmonary Complication; RF = Random Forest; SVM = Support Vector Machine; TJA = Total Joint Arthroplasty; TV = Temporal Validation; VTE = Venous Thromboembolism; WEKA = Waikato Environment for Knowledge Analysis (machine learning workbench developed at the University of Waikato, New Zealand); XGB = XGBoost.

Table S8. AI models associated with less studied PPRC phenotypes (postoperative pulmonary edema) across study-listings (1/63)

Author (Year) / Country or DB	Sample size (*n*)	Surgery / Procedure type	AI/ML methodology	TIER/ Outcome definition / Event rate	Validation strategy	Best model & AUROC (or relevant metric)	Key limitation
Kim, J.-H. et al. (2023) [82] / South Korea	Total 256,899 patients from 5 hospitals; 221,908 (derivation, 4 hospitals) / 34,991 (IV [Quasi-EV], 1 hospital)	Mixed surgery	XGB, LGBM, MLP, LR, BRF	•TIER A<br>•Diagnosed during hospitalization by radiologists on the basis of chest radiographs<br>•Event rate:<br>-Derivation: 1.6% (3584/ 221,908)<br>-IV (Quasi-EV): 5.4% (1896/34,991)	Multicenter institutional network, Quasi-external validation (4-vs-1 hospital geographic split within single institutional network sharing same EHR system)	BRF: AUROC 0.91; PR-AUC 0.44	•Geographically limited external validation<br>•Limited interpretability and prospective applicability of the ML model<br>•Discrimination–utility gap<br>•Outcome ascertainment limited to radiologist-confirmed CXR

Sample size is reported as Derivation cohort / Internal Validation (IV) / Internal Temporal Validation (ITV) / External Validation (EV) cohorts, using the format reported in the original publication. For Kim J.H. 2023, the multicenter institutional network was split into 4 hospitals for derivation and 1 hospital for IV (quasi-external validation), all sharing the same EHR system.

Event rate is reported per cohort when separately reported in the original publication. For Kim J.H. 2023 [82], derivation and quasi-EV cohorts showed substantially different event rates (1.6% vs 5.4%), reflecting differences in patient case-mix between the cohorts despite operating within the same institutional network.

Quasi-External Validation (Quasi-EV) denotes validation using a geographically distinct cohort within the same institutional network sharing the same EHR system and clinical practice patterns. This design approximates true cross-institutional external validation but does not provide equivalent evidence of generalizability across independent institutions with potentially different patient populations, clinical practices, and EHR implementations.

Validation strategy classification:

Internal Validation (IV) = validation using held-out portion of derivation cohort via cross-validation or train-test split.

Quasi-External Validation (Quasi-EV) = validation using a geographically distinct cohort within the same institutional network sharing infrastructure (EHR system, clinical practice patterns); approximates but does

not equate to true cross-institutional external validation.

External Validation (EV) = validation using an independent institutional dataset distinct from the derivation cohort and not sharing the same institutional infrastructure.

Abbreviations (alphabetical):

AUROC = Area Under the Receiver Operating Characteristic curve; BRF = Balanced Random Forest (class-imbalance–adjusted random forest classifier suitable for rare-event prediction); CXR = Chest X-ray; DB = Database; EHR = Electronic Health Record; EV = External Validation; ITV = Internal Temporal Validation; IV = Internal Validation; LGBM = Light Gradient Boosting Machine; LR = Logistic Regression; ML = Machine Learning; MLP = Multilayer Perceptron; PR-AUC = Precision-Recall Area Under the Curve (equivalent to AUPRC); Quasi-EV = Quasi-External Validation (geographically distinct cohort within same institutional network sharing same EHR system); TACO = Transfusion-Associated Circulatory Overload; TRALI = Transfusion-Related Acute Lung Injury; XGB = XGBoost.

Table S9. Tier distribution across study-listings (n = 63) by phenotype

Phenotype	N	Tier A	Tier B	Tier C
Combined PPC	10	6 (60%): Chen S. (2025), Peng X. (2022), Li S. (2024), Zhou C.-M. (2024), Li L. (2024), Li P. (2024)	2 (20%): Jing X. (2022), Kim J.-H. (2023)	2 (20%): Xue Q. (2021), Qiu X. (2025)
Pneumonia/Respiratory infection (Total)	21	8 (38.1%): Xiang B. (2024), Chen C. (2021), Li X. (2024), Bonde A. (2021), Guo J. (2023), Lee T. (2023), Luo Y. (2020), Wang T. (2024)	11 (52.4%): Tang M. (2025), Ma J. (2025), Liu J. (2025), Dai A. (2024), Cong X. (2025), Kim J.-H. (2023), Hu A. (2025), Chen Y. (2025), Endo T. (2026), Lu C. (2023), Chen B. (2024)	2 (9.5%): Xue B., (2021) Li M.-P. (2023)
Pneumonia	17	8 (47.1%)	7 (41.2%)	2 (11.8%)
Respiratory infection	4	0	3 (75.0%)	1 (25.0%)
Hypoxemia	8	8 (100%): Lundberg S.-M. (2018), Lu W. (2024), Kim J.-W. (2025), Park J.-B. (2023), Fang Z. (2023), Zheng L. (2025), Kang H. (2023), Shin Y. (2023)	0	0
Respiratory failure	12	7 (58.3%): Xu M. (2025), Kiyatkin M.-E. (2023), Zhang H. (2024), Wu W. (2025), Yoon H.-K. (2024), Hofer I.-S. (2020), Bonde A. (2021)	2 (16.7%): Chen B. (2024), Blinder J.-J. (2024)	3 (25%): Bihorac A. (2019), Bolourani S. (2020), Hadaya J. (2022)
PE-inclusive VTE	11	9 (81.8%): Nudel J. (2021), Chen Z. (2025), Shohat N. (2023), Qiao N. (2023), Ali H. (2024), Selby L.-V. (2018), Shi J. (2021), Lex J.-R. (2024), Bonde A. (2021)	0	2 (18.2%): Xue B. (2021), Ding R. (2023)
Less studied PPC phenotypes (postoperative pulmonary edema)	1	1 (100%): Kim, J.-H. (2023)	0	0
Total	63	39 (61.9%)	15 (23.8%)	9 (14.3%)

Outcome ascertainment quality was classified using a tiered framework introduced in this review, grounded in TRIPOD+AI reporting standards and PROBAST risk-of-bias assessment principles. The classification reflects the rigor and standardization of outcome definitions used in each study, with implications for generalizability and cross-study comparability.

TIER classification:

TIER A — Outcome ascertainment based on international consensus criteria (e.g., EPCO, StEP-COMPAC, Berlin ARDS criteria, CDC/NHSN, ATS/IDSA pneumonia guidelines) or direct objective measurement (e.g., peripheral oxygen saturation [SpO_2] for hypoxemia; imaging-confirmed pulmonary embolism on CT pulmonary angiography or deep vein thrombosis on compression ultrasonography; radiologist-confirmed pulmonary edema on chest radiograph; ACS-NSQIP or MBSAQIP registry-standard definitions with imaging confirmation).

TIER B — Outcome ascertainment based on national or society guidelines, or study-specific definitions with explicit operational criteria. Examples include Chinese hospital-acquired pneumonia (HAP)/ventilator-associated pneumonia (VAP) guidelines, study-specific composite respiratory failure definitions with documented component criteria, and structured outcome definitions developed for individual studies that approximate but do not strictly meet TIER A consensus standards.

TIER C — Outcome ascertainment based on administrative codes (e.g., ICD-9-CM, ICD-10 diagnostic codes) without imaging confirmation or independent adjudication, or implicit/insufficiently documented outcome definitions. ICD-based ascertainment may underestimate clinically significant events when coding depends on symptom recognition, may include asymptomatic events of unclear clinical relevance, and is susceptible to coding variability across institutions and over time.

Abbreviations (alphabetical):

ACS-NSQIP = American College of Surgeons National Surgical Quality Improvement Program; ARDS = Acute Respiratory Distress Syndrome; ATS = American Thoracic Society; CDC = Centers for Disease Control and Prevention; CT = Computed Tomography; CXR = Chest X-ray; EPCO = European Perioperative Clinical Outcome (consensus framework for postoperative pulmonary complication definitions); HAP = Hospital-Acquired Pneumonia; ICD-9-CM = International Classification of Diseases, 9th Revision, Clinical Modification; ICD-10 = International Classification of Diseases, 10th Revision; IDSA = Infectious Diseases Society of America; MBSAQIP = Metabolic and Bariatric Surgery Accreditation and Quality Improvement Program; NHSN = National Healthcare Safety Network; PE = Pulmonary Embolism; PE-inclusive VTE = Venous Thromboembolism endpoint inclusive of PE alone and PE+DVT composite outcomes; PPC = Postoperative Pulmonary Complication; PROBAST = Prediction model Risk Of Bias ASsessment Tool; SpO_2 = Peripheral oxygen saturation; StEP-COMPAC = Standardised Endpoints in Perioperative Medicine — Postoperative Pulmonary Complications; TIER = Tiered framework for outcome ascertainment quality (introduced in this review); TRIPOD+AI = Transparent Reporting of a multivariable prediction model for Individual Prognosis Or Diagnosis — extension for Artificial Intelligence; VAP = Ventilator-Associated Pneumonia; VTE = Venous Thromboembolism.

Table S10. Descriptive comparison of internal and external validation performance among PPRC AI/ML study-listings with cross-institutional external validation

No.	Study	PPRC phenotype	Model selected for comparison	Internal validation setting	Internal AUROC	External validation setting	External AUROC	ΔAUROC*
1	Peng et al., 2022	Combined PPCs	Deep neural network	West China Hospital; five repeated 5-fold CV	0.884	Six other participating hospitals, combined	0.889	−0.005
2	Li et al., 2024	Postoperative pneumonia / respiratory infection	Logistic regression	Single-center internal test set	0.910	MIMIC-IV	0.890	0.020
3	Dai et al., 2024	Postoperative pneumonia / respiratory infection	CatBoost	Single-center internal test set	0.835	Fourth Affiliated Hospital of Nanjing Medical University	0.894	−0.059
4	Bonde et al., 2021	Postoperative pneumonia / respiratory infection	Deep neural network	ACS-NSQIP validation set	0.885	Independent single-center test set	0.872	0.013
5	Fang et al., 2023	Perioperative hypoxemia	ML model	Internal testing cohort	0.947	Independent external-center cohort	-†	Not calculable
6	Zheng et al., 2025	Perioperative hypoxemia	Random forest	Single-center internal test set	0.914	Independent hospital cohort	0.848	0.066
7	Zhang et al., 2024	Respiratory failure / ARDS	Deep Forest	Nanjing First Hospital testing cohort	0.884	Shanghai General Hospital	0.827	0.057
8	Yoon et al., 2024	Respiratory failure	Gradient boosting	SNUH internal test set	0.912	Three independent hospital cohorts	0.894‡	0.018‡
9	Bonde et al., 2021	Respiratory failure	Deep neural network	ACS-NSQIP validation set	Unplanned intubation: 0.914; prolonged ventilation >48: 0.952	Independent single-center test set	Unplanned intubation: 0.902; prolonged ventilation >48: 0.935	Unplanned intubation: 0.012; prolonged ventilation >48: 0.017
10	Bonde et al., 2021	Pulmonary embolism / VTE	Deep neural network	ACS-NSQIP validation set	0.813	Independent single-center test set	0.763	0.05
11	Shi et al., 2021	Pulmonary embolism / VTE	EasyCIE-PEDVT	University of Utah internal validation cohort	PE: 0.854; DVT: 0.920	Independent healthcare system	PE: 0.907; DVT: 0.921	PE: −0.053; DVT: −0.001

* ΔAUROC (Area Under the Receiver Operating Characteristic curve) = internal AUROC − external AUROC. Positive values indicate lower discrimination in external validation; negative values indicate higher discrimination in external validation.

† Studies were included in the internal–external AUROC difference calculation only when the same model had both internal and external AUROC values available.
‡ For Yoon et al., the three external AUROCs were 0.879, 0.872, and 0.931; the value shown is their unweighted arithmetic mean, **0.894**.

Table S11. Distribution of algorithm by phenotype

Phenotype	*n*	Tree-based	LR/Linear	Deep Learning	Other
Combined PPCs	10	40.0%	30.0%	20.0%	10.0% (Stacking)
Pneumonia / Pulmonary infection	21	52.4%	23.8%	23.8%	0%
Pneumonia	*17*	*47.1%*	*23.5%*	*29.4%*	*0%*
Pulmonary infection	*4*	*75.0%*	*25.0%*	*0%*	*0%*
Hypoxemia	8	75.0%	0%	0%	25.0% (Hybrid/Mixed)
Respiratory failure	12	66.7%	0%	25.0%	8.3% (Ensemble)
PE-inclusive VTE	11	36.4%	27.3%	18.2%	18.2% (NLP)
Less studied PPC phenotypes (postoperative pulmonary edema)	1	100% (BRF)	0%	0%	0%
TOTAL	63	54.0%	17.5%	19.0%	9.5%

Algorithm distribution by phenotype reflects the best-performing model reported in each of the 63 study-listings included in this review. When multiple algorithms were evaluated in a single study (typical for most studies), classification was based on the model selected by the authors as the final or best-performing model with the highest discrimination metric (typically AUROC). For studies using time-stratified or composite approaches with multiple best models per sub-task (e.g., Fang Z. 2023 [53] with separate best models for induction vs intraoperative phases), the study was classified under "Hybrid/Mixed" in the Other category.

Algorithm category definitions:

Tree-based — Models based on decision tree architectures, including:

- Single decision tree (DT, CART)
- Random Forest (RF), Balanced Random Forest (BRF)
- Gradient boosting families: Gradient Boosting Machine (GBM), Gradient Boosting Decision Tree (GBDT), Gradient Boosted Trees (GBT), XGBoost (XGB), Light Gradient Boosting Machine (LGBM), CatBoost
- AdaBoost (AdaB)
- Composite tree-based platforms (e.g., MySurgeryRisk, Bihorac 2019 [57])
- Deep Forest (DF; multi-layer ensemble of random forests — classification varies; in this table classified under DL given its deep architectural design)

LR/Linear — Linear models with explicit interpretability, including:

- Logistic Regression (LR), L2-regularized LR (Ridge), LASSO-regularized LR
- Linear Discriminant Analysis (LDA)
- Generalized Linear Model (GLM), boosted GLM (glmboost)
- Partial Least Squares Discriminant Analysis (PLS-DA)
- LR-based nomograms (commonly used for clinical deployment)

Deep Learning (DL) — Neural network architectures with multiple hidden layers, including:

- Deep Neural Network (DNN), Artificial Neural Network (ANN), Multilayer Perceptron (MLP)
- Convolutional Neural Network (CNN)
- Long Short-Term Memory (LSTM) recurrent neural networks
- Transformer architectures
- Probabilistic Bayesian Neural Network (PBNN)

Other — Methods not falling into the above three categories:

- NLP-based approaches using natural language processing for outcome ascertainment or risk prediction (Selby L.V. 2018 [10] with WEKA bag-of-words; Shi J. 2021 [80] with EasyCIE-PEDVT)
- Hybrid/Mixed approaches combining multiple algorithm families per sub-task (Fang Z. 2023 [53] time-stratified 4-model; Lu W. 2024 [52] stacking classifier combining LR+SVM)
- Stacking/Ensemble approaches combining heterogeneous base learners via meta-learners (Chen S. 2025 [26] in Combined PPCs; Qiao N. 2023 [79] ensemble in PE/VTE)

Abbreviations (alphabetical):

AdaB = AdaBoost; ANN = Artificial Neural Network; ATS = American Thoracic Society; AUROC = Area Under the Receiver Operating Characteristic curve; BiPAP = Bilevel Positive Airway Pressure; BRF = Balanced Random Forest (class-imbalance–adjusted random forest classifier); CART = Classification And Regression Tree; CatBoost = Categorical Boosting; CDC = Centers for Disease Control and Prevention; CNN = Convolutional Neural Network; DF = Deep Forest (multi-layer ensemble of random forests); DL = Deep Learning; DNN = Deep Neural Network; DT = Decision Tree; EasyCIE-PEDVT = Easy Clinical Information Extractor configured for PE and DVT detection ; EHR = Electronic Health Record; EPCO = European Perioperative Clinical Outcome (consensus framework); GBDT = Gradient Boosting Decision Tree; GBM = Gradient Boosting Machine; GBT = Gradient Boosting Tree; glmboost = Boosted Generalized Linear Model; HAP = Hospital-Acquired Pneumonia; HFNC = High-Flow Nasal Cannula; Hybrid/Mixed = combinations of multiple algorithm families per sub-task; LASSO = Least Absolute Shrinkage and Selection Operator; LDA = Linear Discriminant Analysis; LGBM = Light Gradient Boosting Machine; LR = Logistic Regression; LSTM = Long Short-Term Memory; MLP = Multilayer Perceptron; MySurgeryRisk = composite predictive analytics platform using random forest as primary classifier; NLP = Natural Language Processing; PBNN = Probabilistic Bayesian Neural Network; PE-inclusive VTE = Venous Thromboembolism endpoint inclusive of PE alone and PE+DVT composite outcomes; PLS-DA = Partial Least Squares Discriminant Analysis; PPC = Postoperative Pulmonary Complication; RF = Random Forest; Stacking classifier = ensemble method combining predictions from multiple base learners via a meta-learner; SVM = Support Vector Machine; VAP = Ventilator-Associated Pneumonia; WEKA = Waikato Environment for Knowledge Analysis (machine learning workbench); XGB = XGBoost.

Table S12. Reporting of model evaluation and clinical translation methods across included publications

Domain	Evaluation or method	Study-listings, n/N (%)
Calibration	Calibration plot / curve / belt	26/59 (44.1%)
	Calibration intercept / calibration-in-the-large (CITL)	4/59 (6.8%)
	Calibration slope	4/59 (6.8%)
	Hosmer–Lemeshow test	5/59 (8.5%)
	Brier score	22/59 (37.3%)
Rare-outcome performance	AUPRC / PR-AUC	22/59 (37.3%)
	PPV / Precision	37/59 (62.7%)
	NPV	15/59 (25.4%)
Clinical utility	Decision-curve analysis (DCA)	14/59 (23.7%)
Class imbalance	Explicitly addressed	30/59 (50.8%)
	Oversampling / synthetic sampling only†	12/59 (20.3%)
	Undersampling only†	4/59 (6.8%)
	Class weighting / cost-sensitive learning†	6/59 (10.2%)
	Hybrid over-/undersampling†	8/59 (13.6%)
Clinical translation	Prospective clinical-impact evaluation	0/59 (0%)

Abbreviations:

AUPRC = Area Under the Precision-Recall Curve (equivalent to PR-AUC)

Table S13. Comparison of established clinical AI methodological frameworks and the proposed PPRC-specific implementation framework

Framework	Primary purpose	Main AI lifecycle stage(s) addressed	Key elements relevant to PPRC implementation	Relationship to the present PPRC pathway
**TRIPOD+AI** (Collins et al., 2024)	Transparent and complete reporting of studies that develop, validate, or update clinical prediction models using regression or machine-learning methods	Reporting of model development, validation, and updating studies	Source of data; participants; outcome definition and assessment; predictors; statistical and AI/ML methods; performance measures; model usability and clinical utility	Provides the reporting standard against which candidate models can be appraised. In the present pathway, TRIPOD+AI items help determine whether a published model is described in sufficient detail for an institution to assess its potential transferability, particularly the outcome, predictor, and performance information relevant to model selection.
**PROBAST / PROBAST+AI** (Wolff et al., 2019; Moons et al., 2025)	Assessment of risk of bias and applicability of prediction model studies	Model development and evaluation; evidence appraisal	Participants, predictors, outcome, and analysis, including signaling questions addressing outcome definition, assessment, and analysis	Provides appraisal principles that **informed** the outcome-ascertainment TIER classification used in this review, particularly considerations related to standardized outcome definition and consistent ascertainment. The present work does not conduct or replace a formal PROBAST/PROBAST+AI assessment.
**DECIDE-AI** (Vasey et al., 2022)	Transparent reporting of early-stage, small-scale live clinical evaluation of AI-based decision-support systems	Early prospective clinical evaluation	Intended clinical-use context; algorithm inputs and outputs; human–AI interaction; explainability; training and validation data; prospective data collection; workflow integration; human factors and usability	Provides guidance relevant to the prospective evaluation of a selected PPRC model in its intended clinical environment. In the present pathway, these considerations become particularly relevant during the transition from prospective validation to deployment, including workflow integration and human–AI interaction before wider clinical use.
**FUTURE-AI** (Lekadir et al., 2025)	International consensus guidance for developing, validating, and deploying trustworthy and deployable AI systems in healthcare	Entire lifecycle, from design and development through deployment and monitoring	Fairness, universality, traceability, usability, robustness, explainability; lifecycle-oriented deployment and monitoring	Provides broad governing principles applicable across the AI lifecycle, including traceability and post-deployment monitoring. The present work operationalizes selected principles for institution-level implementation within the specific clinical domain of PPRCs rather than extending these principles.
**Present PPRC pathway** (this work)	Institution-level translation of published PPRC prediction-model evidence into local clinical implementation	① Assessment of institutional readiness and clinical need → ② Model selection → ③ Local adaptation → ④ Validation → ⑤ Deployment → ⑥ Monitoring	PPRC phenotype definition and outcome ascertainment; local data compatibility; phenotype-appropriate model selection; predictor feasibility and local adaptation; staged validation; workflow integration; performance and clinical-utility monitoring	**No individual principle in this pathway is newly developed.** External validation, prospective evaluation, local adaptation, workflow integration, fairness, and monitoring are addressed by existing guidance. The contribution of the present work is their **PPRC-specific operationalization**: it makes phenotype definition and outcome ascertainment explicit considerations because PPRC phenotypes and their ascertainment methods are heterogeneous; links model selection and adaptation to an institution's local phenotype, data, and ascertainment capability; and sequences these established principles into a practical institution-level implementation pathway.

Abbreviations: AI, artificial intelligence; DECIDE-AI, Developmental and Exploratory Clinical Investigations of DEcision support systems driven by Artificial Intelligence; FUTURE-AI, Fairness, Universality, Traceability, Usability, Robustness, and Explainability for healthcare AI; ML, machine learning; PPRC, perioperative pulmonary and respiratory complication(s); PROBAST, Prediction model Risk Of Bias ASsessment Tool; PROBAST+AI, Prediction model Risk Of Bias ASsessment Tool for models using regression or artificial intelligence; TRIPOD+AI, Transparent Reporting of a multivariable prediction model for Individual Prognosis Or Diagnosis plus Artificial Intelligence.
